# Modes of Action of a Novel c-MYC Inhibiting 1,2,4-Oxadiazole Derivative in Leukemia and Breast Cancer Cells

**DOI:** 10.3390/molecules28155658

**Published:** 2023-07-26

**Authors:** Min Zhou, Joelle C. Boulos, Ejlal A. Omer, Sabine M. Klauck, Thomas Efferth

**Affiliations:** 1 Department of Pharmaceutical Biology, Institute of Pharmaceutical and Biomedical Sciences, Johannes Gutenberg University-Mainz, Staudinger Weg 5, 55128 Mainz, Germany; 2 Division of Cancer Genome Research, German Cancer Research Center (DKFZ), German Cancer Consortium (DKTK), National Center for Tumor Disease (NCT), Im Neuenheimer Feld 460, 69120 Heidelberg, Germany

**Keywords:** 1,2,4-oxadiazole, c-MYC inhibitor, leukemia, natural product derivative, oncogenes, triple-negative breast cancer

## Abstract

The *c-MYC* oncogene regulates multiple cellular activities and is a potent driver of many highly aggressive human cancers, such as leukemia and triple-negative breast cancer. The oxadiazole class of compounds has gained increasing interest for its anticancer activities. The aim of this study was to investigate the molecular modes of action of a 1,2,4-oxadiazole derivative (ZINC15675948) as a c-MYC inhibitor. ZINC15675948 displayed profound cytotoxicity at the nanomolar range in CCRF-CEM leukemia and MDA-MB-231-pcDNA3 breast cancer cells. Multidrug-resistant sublines thereof (i.e., CEM/ADR5000 and MDA-MB-231-BCRP) were moderately cross-resistant to this compound (<10-fold). Molecular docking and microscale thermophoresis revealed a strong binding of ZINC15675948 to c-MYC by interacting close to the c-MYC/MAX interface. A c-MYC reporter assay demonstrated that ZINC15675948 inhibited c-MYC activity. Western blotting and qRT-PCR showed that c-MYC expression was downregulated by ZINC15675948. Applying microarray hybridization and signaling pathway analyses, ZINC15675948 affected signaling routes downstream of c-MYC in both leukemia and breast cancer cells as demonstrated by the induction of DNA damage using single cell gel electrophoresis (alkaline comet assay) and induction of apoptosis using flow cytometry. ZINC15675948 also caused G2/M phase and S phase arrest in CCRF-CEM cells and MDA-MB-231-pcDNA3 cells, respectively, accompanied by the downregulation of CDK1 and p-CDK2 expression using western blotting. Autophagy induction was observed in CCRF-CEM cells but not MDA-MB-231-pcDNA3 cells. Furthermore, microarray-based mRNA expression profiling indicated that ZINC15675948 may target c-MYC-regulated ubiquitination, since the novel ubiquitin ligase (ELL2) was upregulated in the absence of c-MYC expression. We propose that ZINC15675948 is a promising natural product-derived compound targeting c-MYC in c-MYC-driven cancers through DNA damage, cell cycle arrest, and apoptosis.

## 1. Introduction

Cancer is mainly caused by genomic alterations that result from the loss of tumor suppressor genes and the activation of oncogenes [1]. The *MYC* gene is one of the oncogenes that has been described as a master regulator of gene expression in multiple biological processes [2,3]. This gene encodes a family of basic helix-loop-helix zipper (bHLHZip) proteins consisting of c-MYC, N-MYC, and L-MYC [4]. MYC acts as a transcription factor that binds to its obligatory partner, MYC-associated factor X (MAX). The MYC-MAX heterodimer activates a large number of genes by binding to E box sequences (5′-CACGTG-3′) within gene promoters and enhancers [5].

MYC-regulated transcription is tightly controlled in non-transformed cells. However, MYC is estimated to be overexpressed up to 70% in various human cancer types [6]. MYC contributes to several hallmarks of cancer, including the escape from programmed cell death, promoting sustainable proliferation, genome instability, escape from immunosurveillance, and change of cellular metabolism [7,8]. The major mechanisms of MYC deregulation are gene alternation and the activation of upstream MYC-related signaling pathways (e.g., NOTCH, WNT, and EGFR) [9]. Moreover, MYC is an unstable protein with a short half-life [10], and its stability is also regulated by post-translational modifications [11]. For example, *MYC* gene amplification largely drives breast carcinogenesis [12], and the chromosomal translocation of the *MYC* gene contributes to the development of T-cell acute leukemia [13]. In mice with MYC-induced hematological cancers, an inactivation of the *MYC* transgene led to tumor regression [14]. To date, there is no approved MYC inhibitor, despite several promising MYC inhibitors being investigated in preclinical or clinical studies [8]. Therefore, there is an ongoing quest for novel effective MYC inhibitors for an improvement of cancer therapy with targeted drugs.

Natural products are a promising resource for drug discovery [15]. Oxadiazole represents a five-membered heterocyclic scaffold with one oxygen and two nitrogen atoms. Oxadiazole-based compounds are a rapidly growing field in drug development. They are fundamental pharmacophores due to their stability in aqueous medium and are commonly employed as bioisosteric substitutes [16,17]. Based on the position of oxygen and nitrogen in the ring, oxadiazoles are classified into four regioisomeric structures [18]. In addition, 1,2,4-Oxadiazoles received considerable attention as witnessed by increasing numbers of published studies [19]. The first natural products from the class of 1,2,4-oxadiazoles were phidianidine A and B that were isolated from marine mollusk *Phidiana militaris* by Alder and Hancock [20]. Interestingly, phidianidine A was identified as an antifoulant agent which is a chemical defense of slow-moving marine organisms to deter predators, other colonizers, or competitors [21]. In the past two decades, a great number of 1,2,4-oxadiazole derivates were synthesized and studied for their pharmacological properties, which exhibit numerous bioactivities, including anti-cancer [22,23], anti-inflammatory [24], anti-bacterial [25], anti-viral [26], anti-malarial [27], anti-diabetic [28], and anti-Alzheimer activities [29]. Zibotentan represents an example of an oxadiazole that reached a clinical phase III trial for the treatment of hormone-resistant prostate carcinoma [30]. Studies revealed that 1,2,4-oxadiazoles derivatives exhibited more powerful anticancer activities than established drugs (e.g., doxorubicin and etoposide) by inhibiting cell proliferation which make them appealing as prospective therapeutic candidates [16,18]. However, the modes of action of 1,2,4-oxadiazoles derivatives in cancer have not been fully understood yet.

Breast cancer is the most common malignancy in females worldwide. Triple-negative breast cancer (TNBC) is characterized by the absence of estrogen (ER)/progesterone (PR) expression and human epidermal growth factor receptor-2 (HER2) amplification [31]. MYC is remarkably elevated in TNBC compared with other breast cancer subtypes. TNBC is highly invasive leading to a poor five-year survival rate and distant recurrence rates [12,32]. On the other hand, acute lymphoblastic leukemia (ALL) is an aggressive hematological cancer frequently appearing in children and adolescents. A multitude of evidence supports the important role of MYC in the initiation and progression of ALL [33]. 

The aim of this study was to investigate the 1,2,4-oxadiazoles derivative ZINC15675948 as a novel c-MYC inhibitor. We determined the anticancer activity of ZINC15675948 against drug-sensitive and -resistant leukemia (CCRF-CEM and CEM/ADR5000) and triple-negative breast cancer (MDA-MB-231-pcDNA3 and MDA-MB-BCRP) cell lines. The effects of ZINC15675948 were studied by using western blot, qRT-PCR, molecular docking, microscale thermophoresis, and c-MYC reporter assay. By means of microarray hybridization and Ingenuity Pathway Analysis, we explored the underlying modes of action of ZINC15675948 regarding cell cycle, apoptosis, autophagy, and DNA damage. In addition, the interactions of ZINC15675948 with the multidrug-resistance-mediating ATP-binding cassette transporters’ P-glycoprotein and breast cancer resistance protein were investigated using molecular docking.

## 2. Results

### 2.1. Growth Inhibition Assay

**Figure 1:**
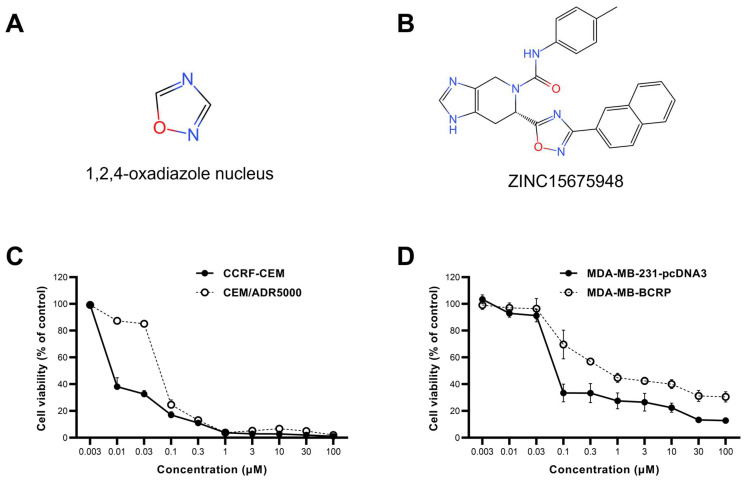
The chemical structure and dose-response curve of ZINC15675948 determined by using the resazurin reduction assay. (**A**) 1,2,4-Oxadiazole nucleus. (**B**) Chemical structure of ZINC15675948. (**C**) Growth inhibition of ZINC15675948 toward leukemia CCRF-CEM and P-glycoprotein-overexpressing CEM/ADR5000 cell lines. (**D**) Growth inhibition of ZINC15675948 toward triple-negative breast cancer MDA-MB-231-pcDNA3 and BCRP overexpressing MDA-MB-BCRP cell lines. The data were plotted as mean ± SD from three independent experiments with each of the 6 parallel measurements.

ZINC15675948 exhibited profound cytotoxicity toward both leukemia and breast cancer cell lines using resazurin reduction assays. Doxorubicin was used as a positive control [34]. CCRF-CEM and MDA-MB-231-pcDNA3 cells were extremely sensitive to ZINC15675948 with IC_50_ values of 0.008 ± 0.001 µM, and 0.08 ± 0.004 µM, respectively. It is interesting that CEM/ADR5000 cells as well as MDA-MB-BCRP cells displayed cross-resistance toward ZINC15675948 (degrees of resistance: 8.37 and 9.0, respectively). The dose-response curves are shown in Figure 1C,D, and the IC_50_ values and degrees of resistance are presented in Table 1.

As multidrug-resistant cell lines were cross-resistant to ZINC15675948, further investigations using ZINC15675948 were performed only in CCRF-CEM and MDA-MB-231-pcDNA3 cells. 

### 2.2. Molecular Docking

**Figure 2:**
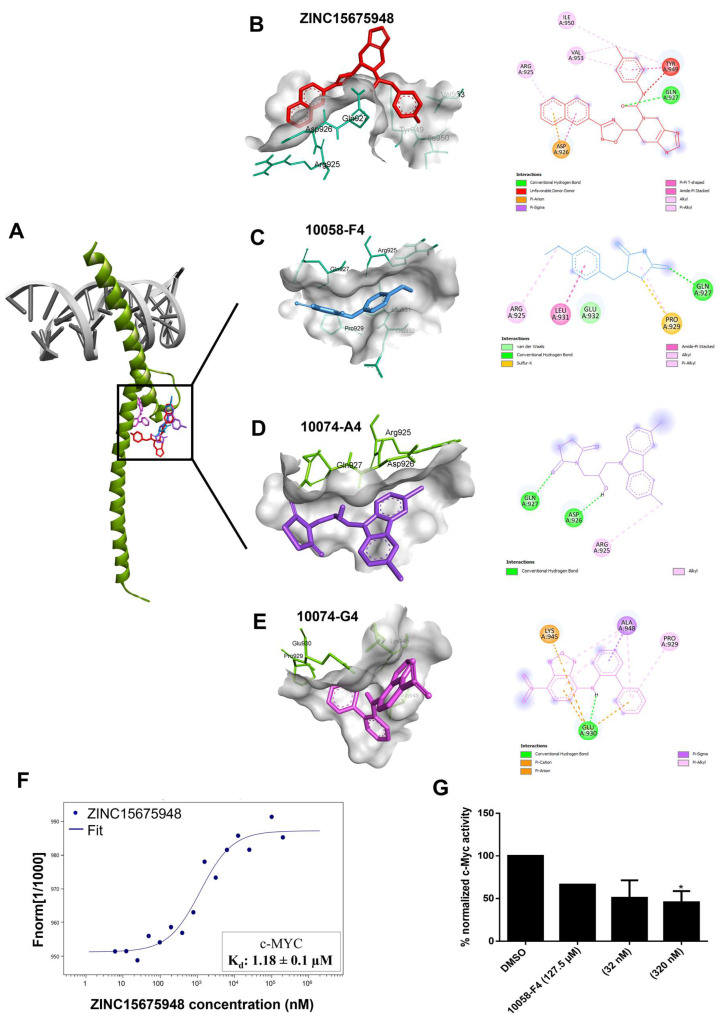
Inhibition of c-MYC by ZINC15675948. (**A**) In silico molecular docking of ZINC15675948 and three known inhibitors 100F4-58, 10074-A4, and 10074-G5 to c-MYC. (**B**) Interacting amino acids of ZINC15675948 (red), (**C**) 10058-F4 (blue), (**D**) 10074-A4 (purple), (**E**) 10074-G4 (violet) interacting with c-MYC as visualized by Discovery Studio. (**F**) Binding kinetics of ZINC15675948 bound to c-MYC obtained by microscale thermophoresis. (**G**) Inhibition of c-MYC activity by ZINC15675948 as determined by a c-MYC reporter assay. Statistical significance (* *p* ≤ 0.05) was compared to DMSO (negative control). All experiments were performed three times independently.

The oncogene *MYC* is deregulated in various human cancers and drives several cancer-related hallmarks. Despite its unquestionable contribution to cancer development, MYC has been regarded as undruggable, and there are only a few MYC inhibitors so far [35,36]. The aim of this study was targeting c-MYC by ZINC15675948, as demonstrated below with different in vitro verifications and pathway analysis of microarray data. To investigate the possible interaction and binding affinity of ZINC15675948 to c-MYC, molecular docking was performed using AutoDock 4.2.6. As shown in Figure 2A, the binding site of ZINC15675948 bound to c-MYC was almost the same as for the known MYC-inhibiting control drugs. ZINC15675948 was particularly near to 10058-F4 and 10074-A4, and Arg925 and Gln927 were two common amino acid residues shown in the interactions, and Gln 927 displayed hydrogen-bonding (Figure 2B–D). Table 2 illustrates that ZINC15675948 displayed a strong binding to c-MYC, revealed by a lowest binding energy (LBE) value of −9.91 kcal/mol and a predicted inhibition constant (pKi) of 0.05 µM. In comparison, the binding affinities of known c-MYC inhibitors were weak (LBE value of 10058-F4: −4.92 kcal/mol; LBE value of 10074-A4: −6.42 ± 0.01 kcal/mol; LBE value of 10074-G5: −6.96 ± 0.01 kcal/mol).

**Figure 3:**
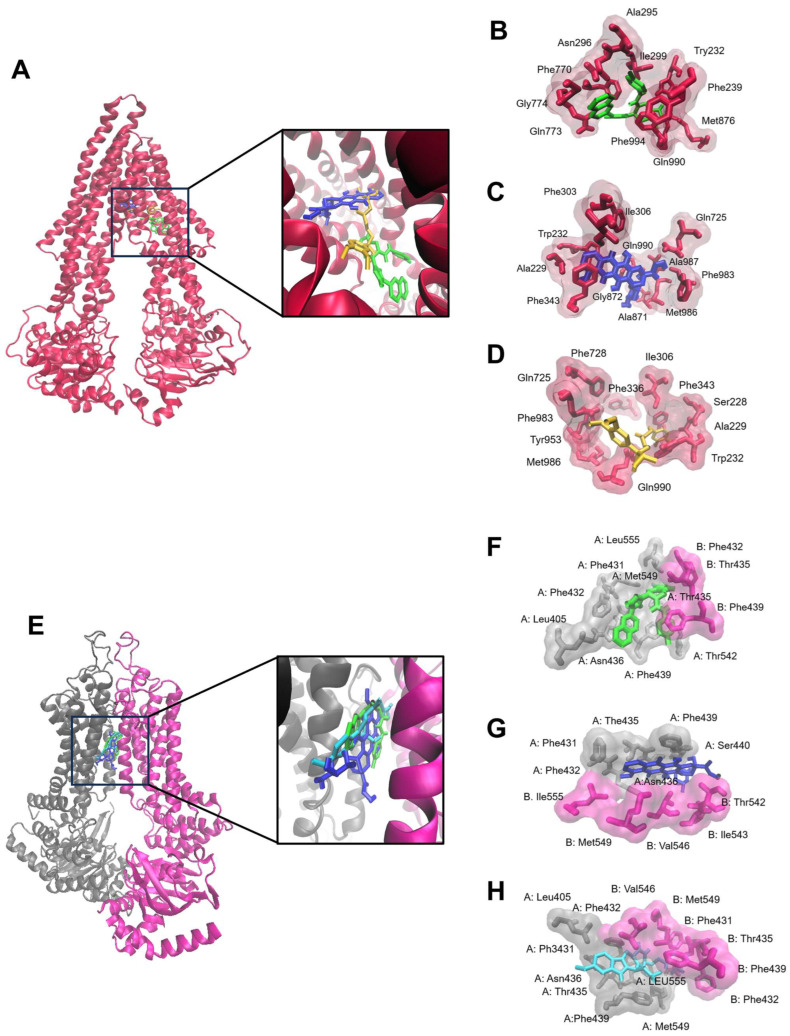
Molecular docking of ZINC15675948 to the ABC transporters (**A**) P-gp and (**E**) BCRP. The proteins were presented in a new carton format. The ligands were displayed using a dynamic bond format with different colors: ZINC15675948 (green), doxorubicin (blue), verapamil (yellow), and Ko143 (cyan). The binding sites were visualized by P-gp residues that interact with (**B**) ZINC15675948, (**C**) doxorubicin, and (**D**) verapamil, as well as BCRP residues that interact with (**F**) ZINC15675948, (**G**) doxorubicin and (**H**) Ko143, are shown in a QuickSurf format.

To better understand the cross-resistance of CCRF-CEM and MDA-MB-231-pcDNA3 cells toward ZINC15675948, molecular docking of ZINC15675948 towards P-gp and BCRP was carried out. Table 3 revealed that ZINC15675948 showed high binding affinities to P-gp and BCRP (LBE values: −10.55 ± 0.24 kcal/mol and −11.49 kcal/mol, respectively). The binding site of ZINC15675948 to P-gp was similar to doxorubicin (Figure 3A). They shared the same amino acid with Trp232 and Gln990 on P-gp (Figure 3B,C). ZINC15675948, likewise doxorubicin, bound to the substrate-binding site on P-gp. However, the binding site of ZINC15675948 was similar to both doxorubicin and Ko143 (Figure 3E–H). We further performed a doxorubicin uptake assay (Section 2.11) to pinpoint whether ZINC15675948 is an inhibitor or substrate of P-gp and BCRP.

### 2.3. Microscale Thermophoresis

To confirm the in silico binding of ZINC15675948 to c-MYC with an in vitro assay, we applied microscale thermophoresis (MST). As shown in Figure 2F, the measurement of the concentration-dependent fluorescence signals revealed an interaction between the fluorescently labeled c-MYC protein and ZINC15675948. ZINC15675948 bound to c-MYC with a K_d_ of 1.08 ± 0.1 µM.

### 2.4. c-MYC Reporter Assay

To determine whether the c-MYC activity could be diminished by ZINC15675948 binding, we performed c-MYC reporter assays in HEK293 cells with a transfected c-MYC-luciferase reporter construct. Notably, the c-MYC activity was suppressed by ZINC15675948 in a concentration-dependent manner. A significant inhibition was observed at a concentration of 320 nM (Figure 2G). Surprisingly, 10058-F4 as a positive control only showed a slight inhibition. Therefore, ZINC15675948 indeed inhibited c-MYC activity as consistently also demonstrated with molecular docking, microscale thermophoresis, and as illustrated below by western blotting and qRT-PCR.

### 2.5. Gene Expression Profile of Cell Lines Using Microarray Analyses

**Figure 4:**
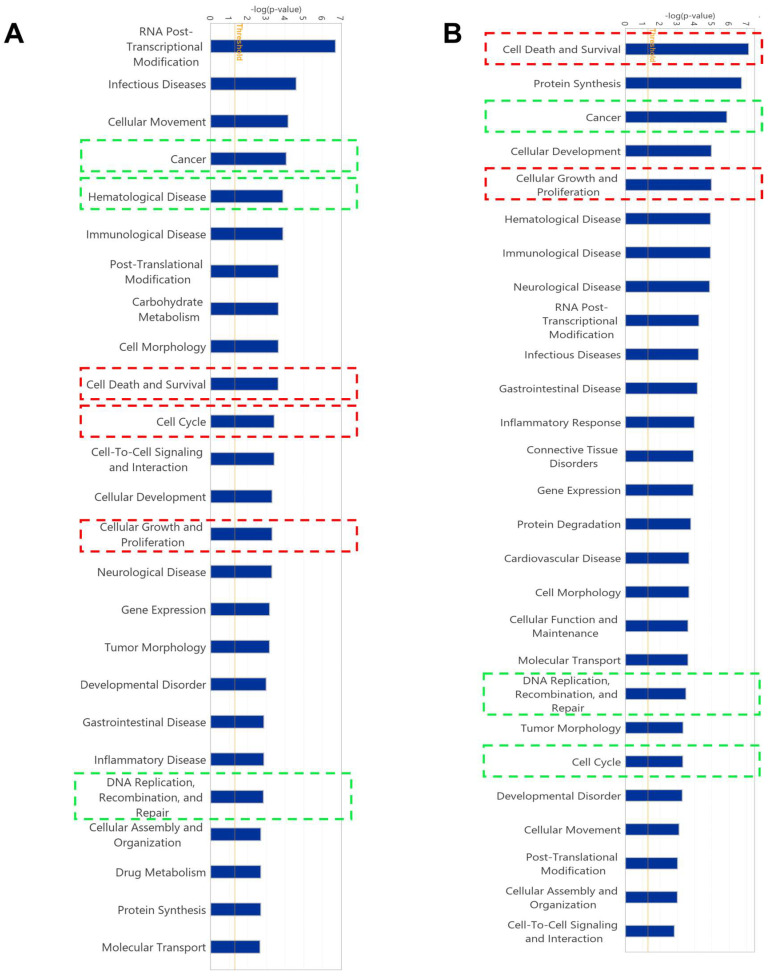
Gene expression profiling as determined by Ingenuity Pathway Analysis (IPA) of CCRF-CEM and MDA-MB-231-pcDNA3 cells upon treatment with the IC_50_ concentration of ZINC15675948 for 24 h. Top cellular functions (red boxes) and diseases (green boxes) affected by ZINC15675948 in (**A**) CCRF-CEM and (**B**) MDA-MB-231-pcDNA3 cells. CCRF−CEM.

The gene expression measured using mRNA microarray hybridization was filtered by Chipster software (version 3.16.3). A total of 329 and 314 genes were significantly deregulated in CCRF-CEM cells and MDA-MB-231-pcDNA3 cells, respectively, compared with their untreated samples (Supplementary Tables S1 and S2). The deregulated genes were further analyzed by Ingenuity Pathway Analysis (IPA) to predict canonical pathways, networks, and cellular functions and diseases affected by ZIN15675948. Here, we did not observe any potentially impacted canonical pathways. Interestingly, several affected cellular functions were commonly revealed in the two cell lines, including “cell death and survival”, “cell cycle”, “cellular growth and proliferation”, as well as “DNA replication, recombination and repair” (Figure 4). “Cancer” and “hematological disease” were affected correspondingly by ZINC15675948 in CCRF-CEM cells. MDA-MB-231-pcDNA3 cells also showed “cancer” as an important affected disease. Therefore, we further investigated the roles of the cell cycle, apoptosis, autophagy, and DNA damage to verify these microarray-based results by independent other methods.

**Figure 5:**
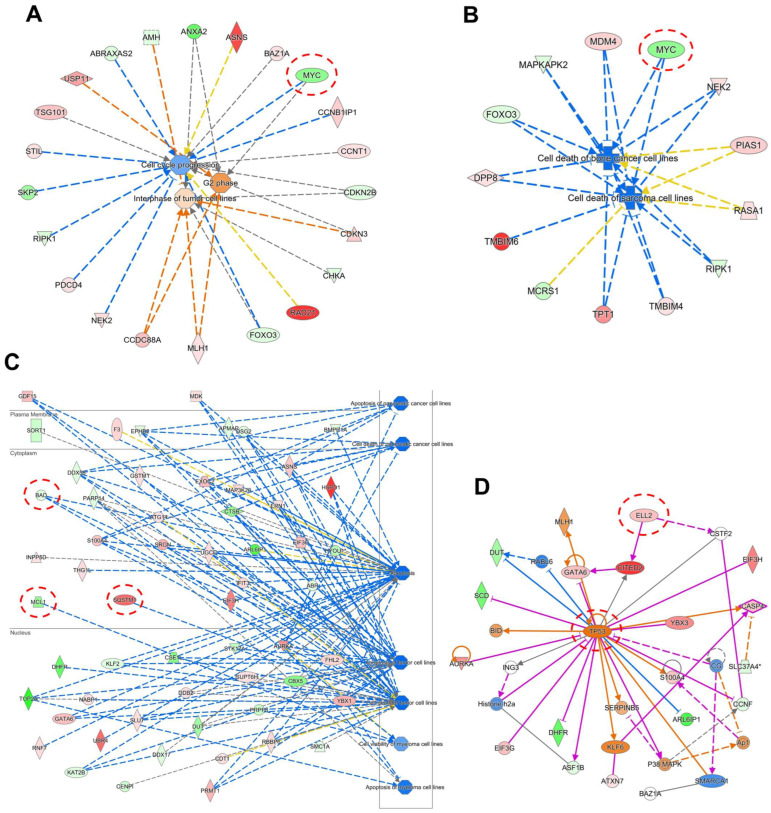
Molecular network generated using IPA software (content version: 51963813) from mRNA microarray hybridization affected by ZINC15675948 in CCRF-CEM and MDA-MB-231-pcDNA3 cells. (**A**) Cell cycle network in CCRF-CEM cells. The red circle highlights that *c-MYC* was downregulated and related to cell cycle regulation. (**B**) Cell death network in CCRF-CEM cells. The red circle highlights that *c-MYC* was also downregulated and involved in cell death. (**C**) Cell cycle network in MDA-MB-231-pcDNA3 cells. The red circles highlight that *SQSTM1* (*p62*) was upregulated, while *MCL-1* and *BAD* were downregulated. (**D**) The red circles highlight that *TP53* and *ELL2* were upregulated in MDA-MB-231-pcDNA3 cells.

We further accessed the networks under different cellular functions to unravel the genes that were involved. The IPA-based comparison of untreated and ZINC15675948-treated CCRF-CEM cells indeed also revealed a downregulation of the *c-MYC* gene as illustrated in Figure 5A,B. These networks are related to cell cycle and cell death, implying that they were downstream and affected by the *c-MYC* gene. However, the *c-MYC* gene did not appear in the gene expression profiles of MDA-MB-231-pcDNA3 cells. The cell death network revealed that *MCL-1* and *BAD* were downregulated accompanied by an upregulation of *SQSTM1* (p62) (Figure 5C). Furthermore, Figure 5D shows that the *ELL2* gene was upregulated, which is involved in the proteasomal degradation of c-MYC [37,38]. The tumor suppressor *TP53* was upregulated, which may be a consequence of *c-MYC* downregulation as we will discuss below. 

### 2.6. Quantitative Reverse Transcription PCR (qRT-PCR)

**Figure 6:**
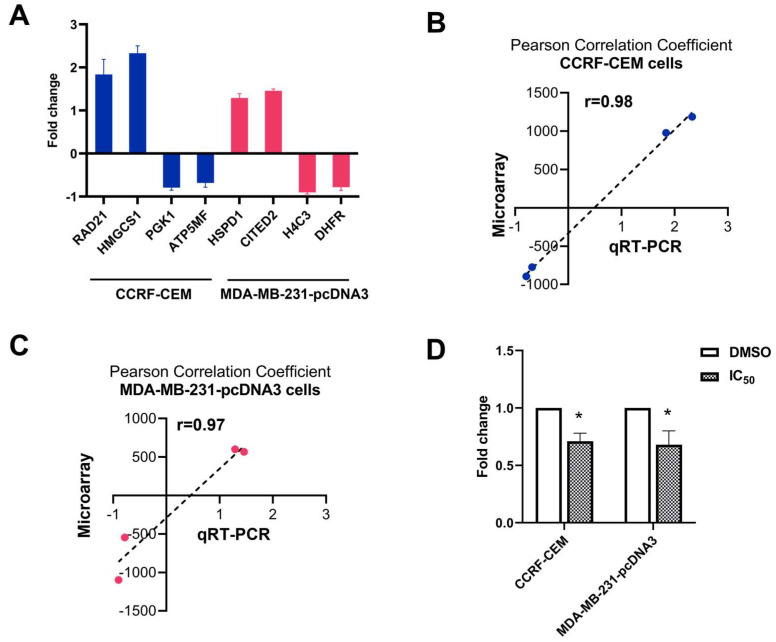
Technical and biological verifications by qRT-PCR analyses in CCRF-CEM and MDA-MB-231-pcDNA3 cells upon treatment with the IC_50_ concentration of ZINC15675948 for 24 h. (**A**) Technical verifications of the top four deregulated genes in CCRF-CEM cells and MDA-MB-231-pcDNA3 cells, respectively. Linear regressions and Pearson correlation coefficients of microarray and qRT-PCR data obtained in (**B**) CCRF-CEM cells and (**C**) MDA-MB-231-pcDNA3 cells. (**D**) Downregulation of *c-MYC* expression in CCRF-CEM and MDA-MB-231-pcDNA3 cells upon treatment with ZINC15675948. Statistical significance (* *p* ≤ 0.05) was compared to control (DMSO). The results are represented as mean values ± SD of three independent experiments.

The technical verifications of the results obtained from microarray hybridization were conducted with the top deregulated genes. Two upregulated and downregulated genes (*RAD21*, *HMGCS1*, *PGK1*, and *ATP5MF*) in CCRF-CEM cells, and two upregulated and downregulated genes (*HSPD1*, *CITED2*, *H4C3*, and *DHFR*) in MDA-MB-231-pcDNA3 cells were subjected to perform qRT-PCR (Figure 6A). The Pearson correlation coefficients were calculated between the determined fold change of microarray hybridization and qRT-PCR data. As shown in Figure 6B,C, the *r* value was 0.98 in CCRF-CEM cells and 0.97 in MDA-MB-231-pcDNA3 cells, confirming a high degree of concordance between these two different methods. Furthermore, the expression level of *c-MYC* was determined by qRT-PCR (Figure 6D). Treatment of ZINC15675948 (IC_50_) significantly downregulated *c-MYC* (*p* ≤ 0.05) in both leukemia and breast cancer cell lines, indicating that ZINC15675948 inhibited *c-MYC* at the gene expression level.

### 2.7. Single Cell Gel Electrophoresis (Alkaline Comet Assay)

**Figure 7:**
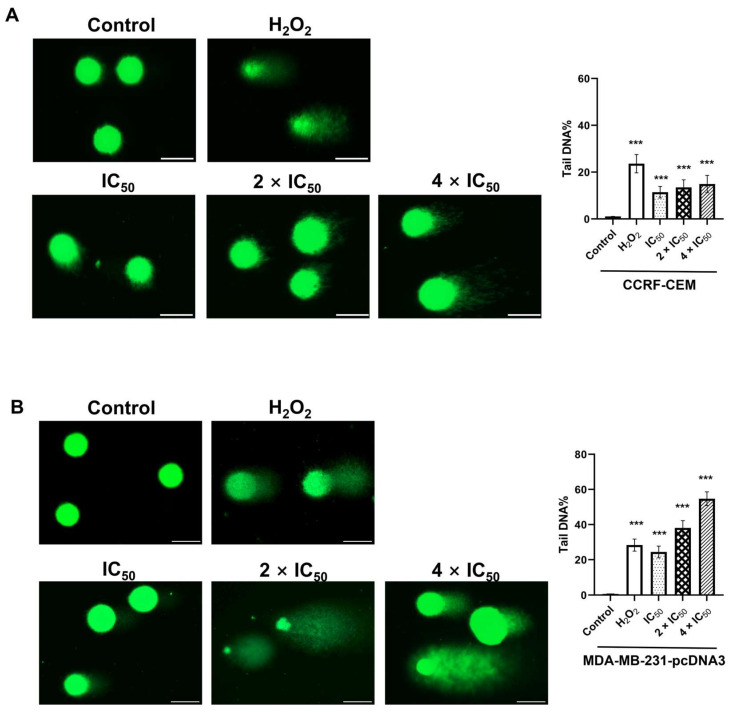
Analysis of DNA damage by single cell gel electrophoreses (alkaline comet assay) induced by ZINC15675948. Representative comet images captured in (**A**) CCRF-CEM and (**B**) MDA-MB-231-pcDNA3 cells treated with different concentrations for 24 h. H_2_O_2_ and DMSO served as positive or negative controls. Scale bar, 50 µm. The graph showed tail DNA percentage presented as mean values ± SEM from 50 comets. Statistical significance (*** *p* ≤ 0.001) was compared to DMSO.

As “DNA replication, recombination and repair” appeared in our IPA analysis as “cellular functions” affected by ZINC15675948, we performed alkaline comet assays to detect DNA damage at the level of single cells. Representative images are shown in Figure 7. Compared with non-damaged control cells (DMSO), there was an increase in ZINC15675948-induced comet tails in both CCRF-CEM and MDA-MB-231-pcDNA3 cells, suggesting that DNA was indeed damaged. H_2_O_2_ as a positive control also led to clearly visible comet tails. The analysis of tails of each 50 cells revealed that ZINC15675948 induced DNA damage in both cell lines in a concentration-dependent manner.

### 2.8. Cell Cycle Arrest

**Figure 8:**
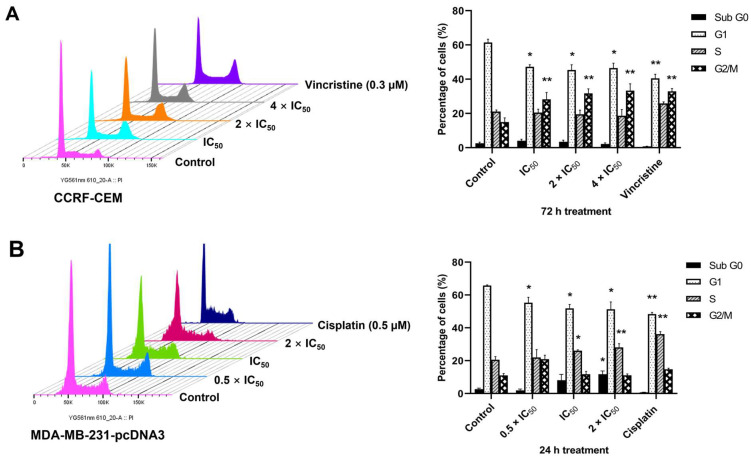
Cell cycle analysis with ZINC15675948. (**A**) Histograms of cell cycle distribution in CCRF-CEM cells upon treatment with different concentrations of ZINC15675948 for 72 h. Vincristine and DMSO were used as positive and negative controls. (**B**) Histograms of cell cycle distribution in MDA-MB-231-pcDNA3 cells upon treatment with ZINC15675948 for 24 h. Cisplatin and DMSO were used as positive and negative controls. The results were represented as mean ± SD from three independent measurements. Statistical significance was analyzed using Student’s *t*-test, * *p* < 0.05, ** *p* < 0.01 compared with DMSO.

The IPA analysis of the microarray data also revealed that cell cycle progression was disturbed by ZINC15675948 in both CCRF-CEM and MDA-MB-231-pcDNA3 cells. Therefore, we investigated the cell cycle distribution by flow cytometry. Figure 8 shows that ZINC15675948 significantly increased the fraction of CCRF-CEM cells in the G2/M phase after treatment for 72 h, which was in a range of 28.25–33.3% at different concentrations compared with DMSO (15.0%). Whereas the S phase fraction of MDA-MB-231-pcDNA3 increased after 24 h. At the highest concentration (2 × IC_50_), S phase arrest reached 28.1% (*p* < 0.01) compared with DMSO as mock control, which was close to the positive control, cisplatin (36.2%) in MDA-MB-231-pcDNA3 cells. Hence, ZINC15675948 arrested the leukemia cells in the G2/M phase and the breast cancer cells in the S phase of the cell cycle.

### 2.9. Detection of Apoptosis

**Figure 9:**
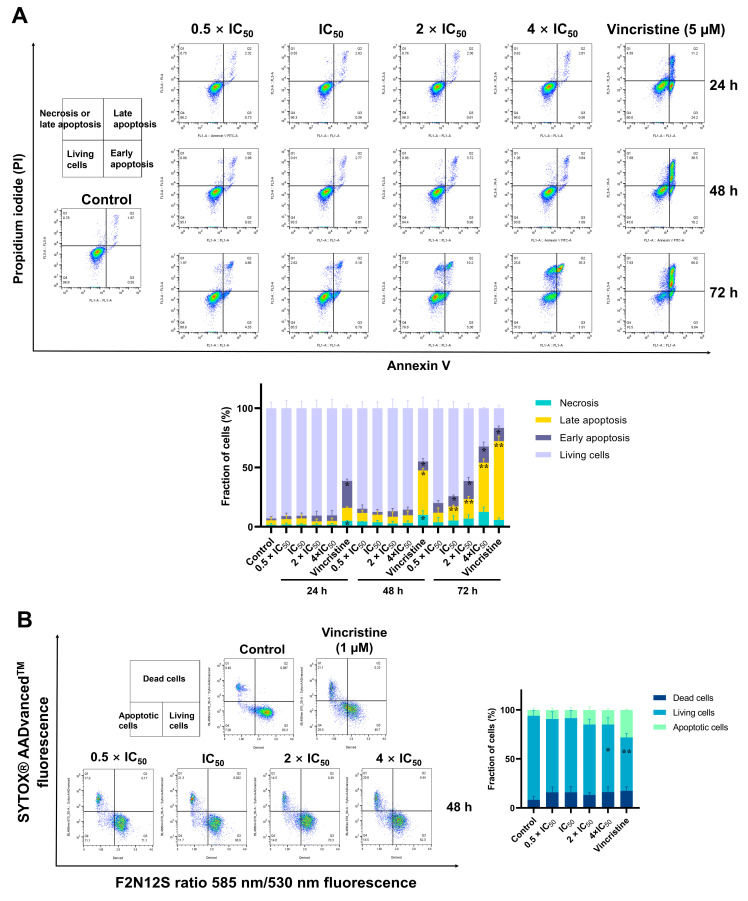
Detection of apoptosis induced by ZINC15675948 by flow cytometry. (**A**) Histograms of CCRF-CEM cells treated with different concentrations of ZINC15675948 for 24, 48, and 72 h. Vincristine (5 µM) and DMSO served as positive and negative controls. Apoptosis was detected using Annexin V and PI staining. (**B**) Histograms of MDA-MB-231-pcDNA3 cells treated with different concentrations of ZINC15675948 for 48 h. Vincristine (1 µM) and DMSO served as positive and negative controls. Apoptosis was determined using F2N12S and SYTOX^®^ AADvancedTM dye. The experiments were performed three times independently. Statistical significance was analyzed using Student’s *t*-test, * *p* < 0.05, ** *p* < 0.01 vs. DMSO.

As “cell death and survival” was revealed as cellular function in the microarray analysis, we measured apoptosis induction by ZINC15675948 using flow cytometry and annexin V and PI double staining in CCRF-CEM cells and with F2N12S and SYTOX^®^ AADvancedTM dead cell staining in MDA-MB-231-pcDNA3 cells. As shown in Figure 9A, ZINC15675948 induced late apoptosis in CCRF-CEM cells in a time- and concentration-dependent manner. The late apoptotic cells remarkably increased to 41.4% at a concentration of 4 × IC_50_ after 72 h, which was similar to the effect of 5 µM vincristine (66%). In parallel, ZINC15675948 also induced apoptosis in MDA-MB-231-pcDNA3 cells after 48 h (Figure 9B). Especially, treatment with 4 × IC_50_ resulted in 14.7% apoptotic cells compared to DMSO (5.9%). Therefore, ZINC15675948 significantly induced apoptosis in both cell lines. 

### 2.10. Western Blotting

**Figure 10:**
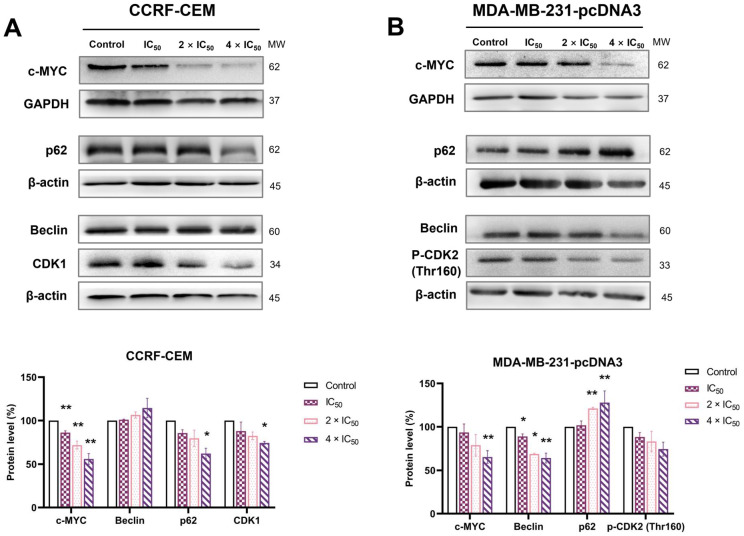
Western blot analysis of c-MYC and proteins involved in cell cycle and autophagy in (**A**) CCRF-CEM and (**B**) MDA-MB-231-pcDNA3 cells treated with various concentrations of ZINC15675948 for 24 h. Bands were normalized to GAPDH or β-actin for quantification (mean ± SEM). Error bars of three repetitions independently were shown. Statistical significance was shown by Student’s *t*-test, * *p* < 0.05, ** *p* < 0.01 compared with DMSO untreated cells.

Western blotting was performed to study the expression of c-MYC and other proteins related to cell cycle and autophagy. CCRF-CEM cells and MDA-MB-231-pcDNA3 cells were treated with ZINC15675948 (IC_50_, 2 × IC_50_ and 4 × IC_50_) for 24 h. Notably, the protein expression of c-MYC was significantly decreased at all concentrations in CCRF-CEM cells (*p* ≤ 0.01). The decrease of c-MYC expression was significant at a concentration of 4 × IC_50_ in MDA-MB-231-pcDNA3 cells (*p* = 0.008) (Figure 10). Therefore, ZINC15675948 inhibited c-MYC expression at the protein level.

To determine whether autophagy contributes to the “cell death and survival” pathway predicted by IPA, we examined two autophagy biomarkers, Beclin and p62. As shown in Figure 10A, the expression of p62 was significantly downregulated by ZINC15675948 at a concentration of 4 × IC_50_ in CCRF-CEM cells (*p* = 0.02) with a slightly raised expression of Beclin, while the expression of p62 was significantly upregulated in MDA-MB-231-pcDNA3 cells, which was consistent with *p62* gene upregulation found with microarray hybridization. The expression of Beclin was accordingly downregulated (Figure 10B). Therefore, ZINC15675948 induced autophagy in CCRF-CEM cells but not in MDA-MB-231-pcDNA3 cells. 

Furthermore, we verified biomarkers of the G2/M and S phases of the cell cycle that were arrested by ZINC15675948 in CCRF-CEM cells and MDA-MB-231-pcDNA3 cells, respectively. Indeed, the expression of CDK1 (indicative for G2/M phase arrest) was downregulated in CCRF-CEM cells, and the expression of phosphorylated CDK2 (Thr160) (indicative for S phase arrest) decreased in MDA-MB-231-pcDNA3 cells.

### 2.11. Doxorubicin Uptake Assay

**Figure 11:**
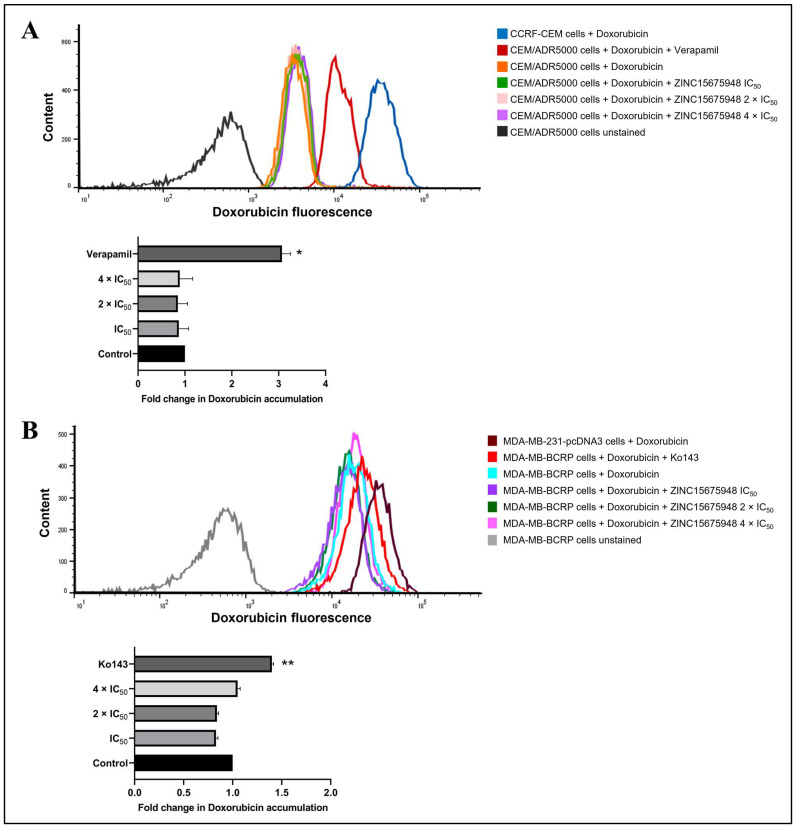
Doxorubicin uptake assay with ZINC15675948. (**A**) Flow cytometric analysis of doxorubicin fluorescence intensity in unstained CEM/ADR5000 cells (autofluorescence, black), CEM/ADR5000 cells treated with doxorubicin (orange), doxorubicin in combination with ZINC15675948 IC_50_ (green), 2 × IC_50_ (pink), and 4 × IC_50_ (violet). CEM/ADR5000 cells treated with doxorubicin in combination with verapamil, a known P-gp inhibitor were used as a positive control. CCRF-CEM cells treated with doxorubicin (blue) were applied as a positive control for doxorubicin uptake. (**B**) Flow cytometric analysis of doxorubicin fluorescence intensity in unstained MDA-MB-BCRP cells (autofluorescence, red-brown), MDA-MB-BCRP cells treated with doxorubicin (cyan), doxorubicin in combination with ZINC15675948 IC_50_ (purple), 2 × IC_50_ (green), and 4 × IC_50_ (pink). MDA-MB-BCRP cells treated with doxorubicin in combination with Ko143 (red), a known BCRP inhibitor was used as a positive control. MDA-MB-231-pcDNA3 cells treated with doxorubicin (grey) were applied as a positive control for doxorubicin uptake. Quantification of fluorescence intensity is shown in the below bar diagram. Three replicates of the experiments were performed, and the results were shown as mean ± SD (* *p* ≤ 0.05, ** *p* ≤ 0.01, compared with doxorubicin-treated cells alone).

Since multidrug-resistant cell lines were cross-resistant to ZINC15675948 in the growth inhibition assay, the uptake assay of doxorubicin using flow cytometry was further carried out in P-gp-overexpressing (CEM/ADR5000) and BCRP-overexpressing (MDA-MB-BCRP) cells to verify the functional role of ZINC15675948 with P-gp and BCRP. As shown in Figure 11A, treatment with positive control verapamil significantly enhanced the ability of CEM/ADR5000 cells to uptake doxorubicin by 3.07-fold (*p* = 0.02). In comparison, ZINC15675948-treated ADR/CEM5000 cells with three different concentrations did not observe doxorubicin accumulation. In MDA-MB-BCRP cells (Figure 11B), Ko143 as a BCRP inhibitor also remarkably increased doxorubicin uptake (*p* = 0.003), while there was no evidence of ZINC15675948 treatments increasing doxorubicin accumulation in MDA-MB-BCRP cells. Therefore, these results confirmed that ZINC15675948 is a substrate of P-gp and BCRP.

## 3. Discussion

Over a time period from 1946 to 2019, natural products and their derivatives accounted for a majority of all anticancer drugs [39], suggesting the eminent importance of natural products in drug development. Seeking novel c-MYC inhibitors represents an urgent demand as c-MYC broadly initiates cancer development. A c-MYC deregulation is frequently involved in the carcinogenesis of lymphoblastic leukemia and triple-negative breast cancer [12,33]. Recently, compounds harboring an oxadiazole scaffold efficiently blocked c-MYC activity. The well-known c-MYC inhibitors, 10074-G5, and 10074-A4, are excellent examples [40,41]. The aim of this study was to investigate a natural product derivate of 1,2,4-oxadiazole, ZINC15675948, as a potential c-MYC inhibitor and its modes of action in leukemia and breast cancer cells. 

We first demonstrated that ZINC15675948 exhibited profound growth inhibitory activity against leukemia and breast cancer cell lines at nanomolar ranges. Another 1,2,4-oxadiazole derivative with reported strong cytotoxic activities toward leukemia and breast cancer cell lines supported our results [18]. Since CEM/ADR5000 and MDA-MB-BCRP cell lines were cross-resistant to ZINC15675948, we selected the drug-sensitive cell lines CCRF-CEM and MDA-MB-231-pcDNA3 to analyze the molecular mechanisms of this compound. 

Next, we addressed the question of whether ZINC15675948 can inhibit c-MYC activity or c-MYC expression. MYC was previously considered as undruggable [42]. Recently, strategies to directly or indirectly inhibit MYC expression with small molecules showed promising results, such as targeting the MYC-MAX interface or disrupting MYC-MAX DNA binding, inducing MYC degradation by ubiquitination protease system, and targeting upstream regulators of MYC [43,44,45]. In our study, molecular docking revealed that ZINC15675948 displayed even better LBE to c-MYC than the three positive drugs. The binding of ZINC15675948 to c-MYC was close to the MYC-MAX interaction site, similar to 10058-F4, 10074-A4, and 10074-G5, which have been demonstrated to bind to the MYC monomer and inhibit its ability to interact with MAX [46]. It is known that one of the most important properties of oxadiazoles is that they act as hydrogen bond acceptors and thus are stable in water-based medium [19]. Our molecular docking definitely showed that ZINC15675948 interacted with Gln927 as a hydrogen bond. Microscale thermophoresis was correlated well with in silico binding, which indicated that ZINC15675948 bound to c-MYC with a K_d_ value of 1.08 ± 0.1 µM. In comparison, 10074-G5 showed the best lowest binding energy of all known c-MYC inhibitors. It bound to MYC with a K_d_ value of 4.4 µM as reported [47]. Meanwhile, the c-MYC reporter assay confirmed that ZINC15675948 showed a better inhibition activity to c-MYC than 10058-F4. Furthermore, the expression of c-MYC was downregulated by ZINC15675948 determined using western blotting and qRT-PCR. Therefore, ZINC15675948 can inhibit c-MYC expression. ZINC15675948 may directly target c-MYC by interfering with the MYC-MAX dimer and thereby affecting c-MYC activity.

In our microarray analysis, *c-MYC* expression was only downregulated in CCRF-CEM cells but not in MDA-MB-231-pcDNA3 cells by ZINC15675948. However, ZINC15675948 upregulated *ELL2* in MDA-MB-231-pcNDA3 cells. Besides transcriptional and post-transcriptional mechanisms, the stability and activity of c-MYC are regulated by a series of post-translational modifications, including phosphorylation, methylation, acetylation, glycosylation, SUMOylation, proline isomerization, and ubiquitination. The ubiquitination-proteasome pathway is one of the prime mechanisms to degrade c-MYC [10,48]. Several E3 ubiquitin ligases were identified to positively or negatively regulate c-MYC inhibition through degradation [49]. The product of the *ELL* (eleven-nineteen lysine-rich leukemia) gene is a transcription elongation factor that accelerates the transcription elongation process of RNA polymerase II. ELL1 (ELL) is the first identified potential c-MYC suppressor in the ELL family. ELL1 functions as an E3 ubiquitin ligase through a CEYLH region in the C-terminus of the protein and targets c-MYC for proteasomal degradation, which thereby inhibits c-MYC-dependent transcriptional activity and cell proliferation [38]. This CEYLH region shares homology within the ELL family suggesting that ELL2 could also have a similar role as ELL1 in the ubiquitin-proteasomal degradation of c-MYC [37]. Therefore, we assume that ELL2 ubiquitinated c-MYC protein in MDA-MB-231-pcDNA3 cells, explaining why *c-MYC* mRNA was not downregulated in the microarray analysis. It is worth pointing out that the c-MYC activity has been indeed reported to be regulated by ubiquitination [49]. Hence, ZINC15675948 may inhibit c-MYC by ubiquitination in MDA-MB-231-pcDNA3 cells.

c-MYC plays a critical role in regulating cell growth, differentiation, cell cycle, apoptosis, metabolism, angiogenesis, DNA repair, immune response, protein translocation, and stem cell formation [6]. However, c-MYC activation is not sufficient alone to cause carcinogenesis. Key mediators of c-MYC-driven cellular processes such as cell cycle checkpoints, apoptosis regulators, as well as tumor suppressor proteins are also required for tumorigenesis. Therefore, synthetic lethal interactions may provide additional opportunities to indirectly inhibit c-MYC [9]. Our microarray hybridization data showed that the “cell cycle”, “cell death and survival” and “DNA damage” functions were affected both in leukemia and breast cancer cells by ZINC15675948. 

DNA damage is an important mechanism of cancer chemotherapeutics that is associated with cell cycle arrest and apoptosis to limit cancer progression [50]. Using single cell gel electrophoresis (alkaline comet assay), we observed that ZINC15675948 remarkably caused DNA damage as seen in increased comet tails upon treatment. In line with this result, ZINC15675948 arrested CCRF-CEM cells in the G2/M phase and MDA-MB-231-pcDNA3 cells in the S phase of the cell cycle as determined by flow cytometry. These results were further confirmed by a downregulated expression of CDK1 or p-CDK2 (Thr160) using western blotting, respectively. C-MYC overexpression promotes cancer cells to re-enter the cell cycle by activating cyclin-dependent kinases (CDKs) and cyclins [9]. CDK1 represents a core component in mammalian cell division. Inhibition of CDK1 induced c-MYC-dependent apoptosis in lymphoblastoid cell lines [51]. CDK2 is activated in the late G1 phase and continues into the S phase. Activation of CDK2 requires phosphorylation of Thr160 [52]. Hence, we conclude that ZINC15675948 treatment resulted in DNA damage and cell cycle arrest as downstream events of c-MYC inhibition.

ZINC15675948 significantly induced apoptosis in both leukemia and breast cancer cell lines. Induction of cell death in CCRF-CEM cells was also indicated as a c-MYC-dependent event by our IPA analysis due to the downregulated *c-MYC* expression. Although *c-MYC* did not appear in the microarray-based dataset of MDA-MB-231-pcDNA3 cells, *TP53* was upregulated. Evidence for crosstalk of c-MYC and p53 networks showed that c-MYC-induced apoptosis is dependent on p53 stabilization and activation [53]. In addition, the induction of apoptosis in MDA-MB-231-pcDNA3 cells correlated well with the microarray-based IPA prediction that *MCL-1* and *BAD* (*BCL-xL*) were downregulated. MCL-1 and BCL-xL are members of the anti-apoptotic B-cell lymphoma 2 (BCL-2) family. Overexpression of anti-apoptotic genes implies an inhibition of apoptosis and leads to cancer development. A recent study revealed that c-MYC-driven human Burkitt lymphoma cells were eradicated by targeting MCL-1 [54]. Therefore, these data favor the view that ZINC15675948-driven inhibition of c-MYC and related signals caused apoptosis. 

In addition to apoptosis, autophagy is a type II programmed cell death that might also be relevant for the modes of action of ZINC15675948. Autophagy is a mechanism that transports damaged proteins or organelles to lysosomes for degradation, playing important roles in maintaining energy homeostasis and ensuring cellular materials’ quality control [55]. If autophagy is induced, Beclin is recruited to organize the pre-autophagosomal structure that is necessary for autophagosome formation [56]. P62 (SQSTM1) transports ubiquitinated cargoes for autophagic degradation. The activation of autophagy declines the expression of p62 [57]. In this study, we demonstrated that the induction of autophagy by ZINC15675948 was cell line-dependent. ZINC15675948 induced autophagy in CCRF-CEM cells as evidenced by a significantly downregulated expression of p62 and an upregulation of Beclin. Induction of autophagy could result from the suppression of c-MYC. These results are supported by our recent report from our group that artemisinin-induced autophagy followed upon c-MYC inhibition in CCRF-CEM cells [58]. Autophagy induction was not observed in MDA-MB-231-pcDNA3 cells, while the upregulated expression of p62 was correlated with that of microarray data. 

ATP-binding cassette (ABC) transporters are widely expressed in various tissues protecting them from endogenous molecules and xenobiotics. ABC transporters are involved in the efflux of cytotoxic drugs from cancer cells leading to the multidrug resistance (MDR) phenotype [59] with broad cross-resistance profiles to diverse chemotherapeutics without structural or pharmacological commonalities, including *Vinca* alkaloids, taxanes, anthracyclines, epipodophyllotoxins, and others [60]. Human P-glycoprotein and BCRP are well-known ABC transporters mediating MDR. P-gp is encoded by the *ABCB1/MDR1* gene and is expressed in the normal intestine, kidneys, liver, placenta, brain, and adrenal glands. BCRP is encoded by the *ABCG2* gene and is also expressed in many tissues, including breast, colon, liver, bile, and in brain capillaries [61,62,63]. P-gp and BCRP confer anticancer drug resistance and were clinically found to correlate with poor diagnosis and survival of cancer patients [64,65]. Cytotoxic agents displaying degrees of cross-resistance above two can be considered as moderate substrates of ABC transporters, while high degrees of resistance (>1000) can also be observed in MDR cells [66,67]. P-gp overexpressing CEM/ADR5000 cells were 8.37 times more cross-resistant to ZINC15675948, while BCRP overexpressing cells were nine times more resistant to ZINC15675948 compared to their sensitive cell lines, indicating that ZINC15675948 was moderately cross-resistant to P-g and BCRP. Using molecular docking, we observed that ZINC15675948 bound at the substrate binding site on P-gp, but its binding site on BCRP is ambiguous concerning the substrate or inhibitor pockets. In the further doxorubicin uptake assay, our data confirmed that ZINC15675948 failed to block the efflux. Therefore, there was no intracellular accumulation of doxorubicin, which can be taken as a further hint that ZINC15675948 is a substrate of P-gp and BCRP. This finding is consistent with our recent report [68]. Future structure-activity relationship (SAR) investigations of derivatives of ZINC15675948 can be carried out to search for more therapeutically promising P-gp and BCRP inhibitors.

## 4. Materials and Methods

### 4.1. Compounds

The compound ZINC15675948 (IUPAC name: (6S)-N-(4-methylphenyl)-6-(3-naphthalen-2-yl-1,2,4-oxadiazol-5-yl)-3,4,6,7-tetrahydroimidazo [4,5-c]pyridine-5-carboxamide) (C26H22N6O2) was purchased from Glentham Life Sciences (Corsham, United Kingdom) (Ref: 44011662 GLS05772). The structure of the 1,2,4-oxadiazole nucleus and ZINC15675948 is shown (Figure 1A,B). The stock solution was prepared with dimethyl sulfoxide (DMSO) at a concentration of 20 mM and stored at −20 °C until use. Since DMSO was the solvent of the compound, DMSO was used as a negative control in all experiments. DMSO was added at the same volume as the highest concentration of ZINC15675948 in the cells, which was less than 1% of the total medium.

### 4.2. Cell Culture

The cell lines investigated in this study were reported previously [69,70]. The drug-sensitive CCRF-CEM and multidrug-resistant CEM/ADR5000 leukemia cell lines were cultivated in complete RPMI 1640 medium (Invitrogen, Darmstadt, Germany). The two breast cancer cell lines transduced with the control vector (MDA-MB-231-pcDNA3) or with a cDNA for the breast cancer resistance protein BCRP (MDA-MB-231-BCRP clone 23) were maintained in DMEM medium (Invitrogen, Darmstadt, Germany). Both media were supplemented with 10% fetal bovine serum (FBS) (Invitrogen) and 1% penicillin (100 µg/mL)-streptomycin (100 µg/mL) (Invitrogen). Cells were cultured at 37 °C in a humidified air incubator (90%) containing 5% CO_2_. In addition, CEM/ADR5000 cells were continuously treated with 5000 ng/mL doxorubicin every two weeks to maintain the overexpression of P-glycoprotein. The gene expression profiles of CEM/ADR5000 cells were previously described in [71,72,73]. MDA-MB-231-BCRP clone 23 cells were treated with 800 ng/mL geneticin (Sigma-Aldrich, Darmstadt, Germany) every two weeks.

### 4.3. Growth Inhibition Assay

The resazurin reduction assay was used to access the effect of growth inhibition of ZINC15675948 [74]. The non-fluorescent dye resazurin is metabolically converted to the fluorescent dye resorufin by living cells [75]. Briefly, the CCRF-CEM and CEM/ADR5000 suspension cells were seeded into 96-well plates (1 × 10^4^ cells/well) and then directly treated with 10 concentrations of ZINC15675948 in a range of 0.3–100 µM, respectively, in a total volume of 200 µL. MDA-MB-231-pcDNA3 and MDA-MB-231-BCRP clone 23 cells were seeded into 96 well plates (5 × 10^3^ cells/well) overnight and treated in the same series of concentrations as in leukemia cells with ZINC15675948 on the following day. After 72 h incubation, 20 µL of 0.01% resazurin (Promega, Germany) were added to each well and incubated for 4 h at 37 °C. The resazurin fluorescence was detected using an Infinite M2000 Pro^TM^ plate reader (Tecan, Crailsheim, Germany) at Ex/Em = 550 nm/590 nm wavelength. Relative cell viability was calculated in comparison to the DMSO-treated control. The final concentration of DMSO was 0.5%. The growth inhibition was accessed according to the effectiveness in inhibiting cell proliferation by half and was expressed as half-maximal inhibitory concentration (IC_50_) values. All IC_50_ values were expressed as mean ± standard deviation (SD). This experiment was repeated three times independently with six wells for each concentration. The figures were analyzed using GraphPad Prism Software (version 9.0.2) (GraphPad Software Inc., San Diego, CA, USA).

### 4.4. Molecular Docking

In silico molecular docking was performed using the AutoDock 4.2.6 software (The Scripps Research Institute, CA, USA). The protocol was recently described by us [69]. The SDF format of ZINC15675948 was downloaded from ZINC 15 database (https://zinc15.docking.org/ accessed on 20 October 2019). ZINC15675948 was considered to perform molecular docking to P-glycoprotein (P-gp, *MDR1, ABCB1*) and the breast cancer resistance protein (BCRP, *ABCG2*), since growth inhibition of ZINC15675948 was determined in P-gp- or BCRP-overexpressing multidrug-resistant cell lines. The 3D structures of c-MYC (PDB code: 1NKP), P-gp (PDB code: 6QEX) and BCRP (PDB code: 6FFC) were downloaded from the RCSB Protein Data Bank (http://www.rcsb.org/, accessed on 20 October 2019) as PDB files. The known c-MYC inhibitors 10058-F4, 10074-A4 and 10074-G5 were used as positive control drugs to compare their affinity and binding mode with ZINC15675948 [76]. C-MYC was covered with grid box for the defined docking mode. The center of the grid box was set at x = 67.083, y = 64.481, and z = 42.943 with a spacing of 0.397 and with a number of grid points of 68 in x, 64 in y, and 72 in z). Doxorubicin as a known substrate of P-gp and BCRP was used as a control drug. Verapamil and Ko143 are inhibitors of P-gp or BCRP, respectively. Both were also used as control drugs. The grid boxes of P-gp and BCRP were placed around the drug-binding site [74]. Hydrogens were added to each protein structure, missing atoms were checked. The Lamarckian GA (4.2) was applied as an algorithm for 250 runs and 25,000,000 energy evaluations for each cycle. Docking was performed three times independently and the predicted inhibition constants were obtained from the docking log files (dlg). VMD (Visual Molecular Dynamics) software (version 1.9.3) (University of Illinois at Urbana Champaign, Champaign, IL, USA) and Discovery Studio Visualizer software (version v.21.1.0.20298) (Dassault Systems Biovia Corp, San Diego, CA, USA) were used as visualization tools to generate figures and gain a deeper understanding of the binding modes that were calculated from docking.

### 4.5. Microscale Thermophoresis

Microscale thermophoresis (MST) was performed with human recombinant c-MYC protein and ZINC15675948. The protein was purchased from Abcam (Cambridge, UK) with a concentration of 0.5 mg/mL (ab169901). C-MYC was labeled with Monolith Protein Labeling Kit RED-NHS 2nd Generation (Nano Temper Technologies GmbH, Munich, Germany) according to the manufacturer’s instructions. The final concentration of c-MYC after labeling was 595 nM. ZINC15675948 was diluted from 400 µM to a series of concentrations in assay buffer (50 mM Tris buffer (pH 7.4) containing 10 mM MgCl_2_, 150 mM NaCl, and 0.05% Tween-20). The labeled protein and diluted compounds were mixed (1:1). After 30 min incubation in the dark at room temperature, the fluorescence signal was measured on a Monolith NT.115 instrument (Nano Temper Technologies) with Monolith NT.115 standard capillaries. The MST with ZINC15675948 was performed with 70% LED power and 10% MST power. Fitting curved and dissociation constant (K_d_) values were calculated with MO. Affinity Analysis software (version 2.2.4) (Nano Temper Technologies). The measurements were repeated three times independently.

### 4.6. c-MYC Reporter Assay

A signal MYC reporter assay (Qiagen, Germantown, MD, USA) was used to determine the impact of ZINC15675948 on c-MYC activity as we described recently [58]. Briefly, a c-MYC-luciferase reporter construct was transfected into human embryonic kidney HEK293 cells and incubated according to the manufacturer’s instructions. Subsequently, cells were treated with two concentrations (4 × IC_50_ in CCRF-CEM or MDA-MB-231-pcDNA3) of ZINC15675948 (32 nM and 320 nM) and DMSO (negative control) or of the known c-MYC inhibitor 10058-F4 (positive control) for 48 h. A Dual-glo^®^ Luciferase Reporter Assay System (Promega, Madison, WI, USA) was applied for the measurement of c-MYC promoter activity. Renilla and firefly luciferase luminescence were measured using an Infinite M2000 Pro^TM^ plate reader (Tecan, Crailsheim, Germany).

### 4.7. Gene Expression Profiles

Total mRNA was isolated using the InviTrap^®^Spin Universal RNA Mini Kit (Invitek Molecular, Berlin, Germany). CCRF-CEM (1 × 10^6^ cells/well) and MDA-MB-231-pcDNA3 cells (5 × 10^5^ cells/well) were treated with DMSO as a solvent control and ZINC15675948 for 24 h in duplicate. ZINC15675948 was applied with a concentration according to the IC_50_ value (CCRF-CEM cells: 0.008 µM, MDA-MB-231-pcDNA3 cells: 0.08 µM). The RNA concentrations were determined using NanoDrop1000 (PEQLAB, Erlangen, Germany). Afterwards, quality control of total RNA, probe labeling, hybridization, scanning and data analysis of the samples were performed at the Genomics and Proteomics Core Facility of the German Cancer Research Center (DKFZ, Heidelberg, Germany). Affymetrix GeneChips^®^ with human Clariom^TM^ S assays (Affymetrix, Santa Clara, CA, USA) were applied for microarray hybridizations as previously described in detail [34].

### 4.8. Pathway Analysis of Microarray Data

The Chipster software (http://chipster.csc.fi/, accessed on 20 October 2019) (The Finnish IT Center for Science CSC, Espoo, Finland) was used to filter a set of differentially expressed genes acquired from microarray hybridization [77]. The Empirical Bayes *t*-test (*p* < 0.05) was applied to access the deregulated genes between DMSO and ZINC15675948-treated groups (accessed in July 2021). The filtered genes were analyzed with the Ingenuity Pathway Analysis (IPA) software (Qiagen, Redwood City, CA, USA) (content version 51963813) by the core analysis tool to determine the cellular functions and networks affected by drug treatment (accessed in August 2021).

### 4.9. Quantitative Real-Time Reverse Transcription PCR

The same total RNA samples (DMSO control and IC_50_) used for the microarray analyses were also used for qRT-PCR experiments [78]. One microgram RNA was converted to cDNA using the LunaScript^®^ RT SuperMix Kit cDNA Synthesis Kit (New England Bio Labs, Darmstadt, Germany) according to the manufacturer’s instructions. All PCR primers were designed using the NCBI Primer-BLAST (https://www.ncbi.nlm.nih.gov/tools/primer-blast/) website and purchased from Eurofins genomics (Ebersberg, Germany) (https://eurofinsgenomics.eu/en/dna-rna-oligonucleotides/optimised-application-oligos/pcr-primers/) (accessed in September 2021). The primer sequences are shown in Table 4. The *GAPDH* gene served as an internal control. The reaction mixture contained 4 µL master mix (5 × Hot Start Taq EveGreen^®^ qPCR Mix (no Rox) (Axon Labortechnik, Kaiserslautern, Germany), 1 µL forward or reversed primer (250 nM final concentration), 13 µL nuclease-free water (Thermo Fisher), and 1 µL cDNA converted from 300 ng RNA. The qRT-PCR was performed on CFX384 Touch Real-Time PCR Detection System (Bio-Rad, Munich, Germany) using the 384-well plate. The initial denaturation of qRT-PCR was at 95 °C for 10 min followed by 40 cycles including strand separation at 95 °C for 15 s, annealing at 57.5 °C for 40 s and extension at 72 °C for 1 min. CFX Manager Software (version 3.1) was used to generate the C_q_ values. The fold-change of gene expression was calculated using comparative 2^−ΔΔ*C*T^ method as reported in the literature [79]. Specifically, for the DMSO control sample, the data of the target gene are presented as fold change in gene expression normalized to *GAPDH* gene. For the sample treat with the IC_50_ of the compound, the evaluation of 2^−∆∆*C*T^ after normalization to *GAPDH* gene reveals the fold change of the target gene in gene expression relative to the untreated control.

### 4.10. Single Cell Gel Electrophoresis (Alkaline Comet Assay)

DNA damage was detected by alkaline comet assay using the OxiSelect™ Comet Assay Kit (Cell Biolabs/Biocat, Heidelberg, Germany) as described in [78]. The alkaline comet assay is a sensitive method for monitoring the migration of DNA fragments from nuclei under alkaline conditions. It detects DNA single- and double-strand breaks at a cellular level [50]. Briefly, CCRF-CEM cells (1 × 10^6^ cells/well) were seeded into a 6-well plate and treated with different concentrations of ZINC15675948 (IC_50_, 2 × IC_50_ and 4 × IC_50_) and DMSO as a negative control for 24 h. MDA-MB-231-pcDNA3 cells (5 × 10^5^ cells/well) were first seeded for 24 h to allow adhesion and then incubated with the same treatments for 24 h. Both cell lines were treated with H_2_O_2_ (50 µM) as a positive control for 30 min [80]. Cells were harvested and centrifuged at 3000× *g* for 10 min and were suspended in 1 mL cold PBS. Next, 1 × 10^5^ cells were counted and mixed with agarose at 37 °C at a ratio of 1:6 and then spread on a comet slide. The following steps were conducted in the dark. Slides were left at 4 °C for 30 min to solidify and then immersed in pre-chilled lysis solution (NaCl 14.6 g, EDTA solution 20 mL, 10× lysis solution, pH 10.0, fulfill to 100 mL with distilled water, stored at 4 °C) for 1 h at 4 °C. Afterwards, slides were immersed in pre-chilled alkaline electrophoresis solution buffer (NaOH 12 g, EDTA solution 2 mL, fulfill to 100 mL with distilled water, stored at 4 °C) for 40 min. Next, electrophoresis was performed for 20 min at 20 V with alkaline electrophoresis solution buffer. Subsequently, slides were transferred into pre-chilled distilled water for 2 × 5 min for washing, followed by immersion in 70% ethanol for 5 min. After slides were dry, Vista Green DNA dye was diluted in TE buffer (Tris 121.14 mg, EDTA 200 µL, pH 7.5, fulfill to 100 mL distilled water) at a ratio of 1:1000. Then 100 µL diluted Vista Green DNA dye was added to each sample. DNA damage was captured by EVOS digital inverted microscope (Life Technologies GmbH, Darmstadt, Germany). At least 50 comets of each treatment were analyzed by Imaged J software (version 1.53q) using a plugin OpenComet (National University of Singapore, Singapore). The tail DNA percentage was used as a parameter of DNA damage [81,82].

### 4.11. Cell Cycle Arrest

CCRF-CEM cells (1 × 10^6^ cells/well) were seeded into a 6-well-plate and treated with ZINC15675948 at concentrations of IC_50_, 2 × IC_50_ or 4 × IC_50_, DMSO (negative control), or vincristine (positive control, 0.3 µM) (University Hospital Pharmacy, Mainz, Germany) for 72 h. MDA-MB-231-pcDNA3 cells (3 × 10^5^ cells/well) were seeded and on the second day treated with ZINC15675948 at concentrations of 0.5 × IC_50_, IC_50_ or 2 × IC_50_, and DMSO (negative control) and cisplatin (positive control, 0.5 µM) (University Hospital Pharmacy) for 24 h. The cells were harvested and centrifuged with cold PBS (4 °C) twice (1500 rpm for 5 min). Ice-cold ethanol (80%) was used for fixation. Samples were kept at −20 °C at least for 24 h. Afterwards, cells were spun down from the ethanol by centrifugation at 4000 rpm for 10 min and washed twice with cold PBS. Before measurement, the cells were resuspended with 500 µL cold PBS containing 20 g/mL RNase (Roche Diagnostics, Mannheim, Germany) and incubated at room temperature for 30 min, followed by staining with 50 μg/mL propidium iodide (PI) (Sigma-Aldrich). After 15 min incubation in the dark at 4 °C, CCRF-CEM cells were analyzed on a BD Accuri^TM^ C6 Flow Cytometer (Becton-Dickinson, Heidelberg, Germany). MDA-MB-231-pcDNA3 cells were analyzed on a BD LSRFortessa SORP (Becton Dickinson, Heidelberg, Germany). The cells were gated firstly using FSC-A/SSC-A gate in linear scale, 10^4^ cells were recorded. Then doublets were removed using FL2-A/FL2-H gate also in linear scale. The DNA histogram was generated using FL2-A/histogram properties. All the experiments were repeated three times independently. The cell cycle distributions were analyzed by the FlowJo software (version 10.8.1) (Celeza, Olten, Switzerland) [34].

### 4.12. Detection of Apoptosis in Suspension Cells

Annexin V-FITC apoptosis kit (Bio Version/Biocat, Heidelberg, Germany) was applied to detect apoptosis in suspension cells [83]. CCRF-CEM cells (1 × 10^6^ cells/well) were seeded in a 6-well plate, then treated with different concentrations (IC_50_, 2 × IC_50_ or 4 × IC_50_) of ZINC15675948, DMSO (negative control), or vincristine (positive control, 5 µM), and incubated for 24, 48, or 72 h. The cells were harvested, washed with cold PBS and 1 × binding buffer (Bio Version), respectively. Afterwards, the cells were stained with 52.5 µL annexin V master mix (2.5 µL annexin V, 50 µL 1 × binding buffer) (Bio Version), and incubated at 4 °C in the dark for 15 min. Then cells were stained with 440 µL PI master mix (10 µL PI, 430 µL 1 × binding buffer) (Bio Version). The detection was performed on a BD Accuri^TM^ C6 Flow Cytometer (Becton-Dickinson) and 2 × 10^4^ cells were recorded for each sample. Four different cell populations were obtained from flow cytometer, including living cells: annexin (−)/PI (−), early apoptosis: annexin (+)/PI (−), late apoptosis: annexin (+)/PI (+), and necrosis: annexin (−)/PI (+). The data were analyzed using FlowJo software (version 10.8.1) (Celeza). The experiments were repeated in triplicate.

### 4.13. Detection of Apoptosis in Adherent Cells

MDA-MB-231-pcDNA3 adherent cells generally need trypsinization for cell-harvesting, which may result in false positive results by applying the Annexin V-FITC apoptosis kit. Therefore, the Violet Ratiometric Membrane Asymmetry Prob/Dead Cell Apoptosis Kit (Thermo Fisher Scientific, Darmstadt, Germany) was alternatively utilized to detect apoptosis in adherent cells [84]. Furthermore, 4’-N,N-diethylamino-6-(N,N,N dodecyl-methylamino-sulfopropyl)-methyl-3-hydroxyflavone (F2N12S) is a novel fluorescent probe to monitor the lipid composition on the plasma membrane in the early stage of apoptosis. SYTOX^®^ AADvanced^TM^ can pass through the cell membrane only in necrosis or late apoptosis. Briefly, 1 × 10^5^ MDA-MB-231-pcDNA3 cells were seeded in a 6-well plate overnight and then treated with different concentrations (0.5 × IC_50,_ IC_50_, 2 × IC_50_ or 4 × IC_50_) of ZINC1565948, DMSO (negative control), or vincristine (positive control, 1 µM). After the incubation for 48 h, cells were detached using 500 µL Accutase (Thermo Fisher Scientific, Darmstadt, Germany) at room temperature and then washed with 1 mL cold Hank’s balanced salt solution (HBSS, 4 °C) twice. Cells were suspended with 1 mL HBSS. Subsequently, 1 µL F2N12S solution was added to each sample at a final concentration of 200 nM. Another 1 µL SYTOX^®^ AADvanced^TM^ dead cell stain solution was added at a final concentration of 1 µM. Samples were incubated at room temperature for 5 min and then analyzed using a BD LSRFortessa SORP (Becton Dickinson). The F2N12S was excited at 405 nm, and the emissions were collected with orange fluorescence (585/15 bandpass filter) and green fluorescence (530/30 bandpass filter). A ratio of the orange fluorescence channel to the green fluorescence channel was set as a derive parameter. The SYTOX^®^ AADvanced ^TM^ dead cell stain was excited at 488 nm, and the emission was collected with a 670/30 bandpass filter. The detections were repeated three times independently. The data were analyzed by the FlowJo software (version 10.8.1) (Celeza).

### 4.14. Protein Analyses by SDS-PAGE and Immunoblotting

CCRF-CEM cells (6,000,000 cells/flask) and MDA-MB-231-pcDNA3 cells (500,000 cells/well) were treated with varying concentrations of ZINC15675948 (IC_50_, 2 × IC_50_, or 4 × IC_50_) or DMSO as a negative control. After 24 h incubation, the cells were harvested and washed with PBS. Then 100 µL of ice-cold M-PER Mammalian Protein Extraction Reagent (Thermo Fisher Scientific, Darmstadt, Germany) containing 1% Halt Protease Inhibitor Cocktail and phosphatase inhibitor (Thermo Fisher Scientific) were added to each sample. The cell lysis solutions were shaken for 30 min at 4 °C and centrifuged at 14,000× *g* for 15 min at 4 °C. The supernatants were harvested, and the protein concentrations were measured with NanoDrop1000 (PEQLAB, Erlangen, Germany).

Each protein sample of 30 mg was electrophoresed on 10% SDS-polyacrylamide gels and transferred to polyvinylidene difluoride (Ruti^®^-PVDF) membranes (Millipore Corporation, Billerica, MA, USA) at 250 mA for 90 min. The membranes were washed with Tris-buffered saline containing 0.1% Tween-20 (TBST) for 5 min and then blocked in 5% bovine serum albumin in TBST for 1 h at room temperature. Afterward, the membranes were washed with TBST for 3 × 5 min and incubated with diluted primary antibody at 4 °C overnight as follows: c-MYC antibody (1:1000, Cell Signaling Technology, Franfurt a. M., Germany), p62, SQSTM1 polyclonal antibody (1:1000, Proteintech, Planegg-Martinsried, Germany), Beclin 1 polyclonal antibody (1:1000, Proteintech), CDK1 (1:1000, Cell Signaling Technology), Phospho-CDK2 (Thr160) (1:1000, Cell Signaling Technology), GAPDH (1:1000, Cell Signaling Technology), or β-actin (1:1000, Cell Signaling Technology). After washing with TBST for 3 × 5 min, the membranes were incubated with diluted secondary antibody anti-rabbit IgG, HRP-linked antibody (1:2000, Cell Signaling Technology) for 1 h at room temperature. Finally, Horseradish peroxidase (HRP) substrate (Luminate^TM^ Classico, Merck Millipore, Schwalbach, Germany) was added to membranes in the dark. The Alpha Innotech FluorChem Q system (Biozym, Oldendorf, Germany) was used for band detection. The quantification was carried out using Image J software (version 1.53q) (National Institute of Health, Bethesda, MD, USA). All the experiments were repeated at least three times independently.

### 4.15. Doxorubicin Uptake Assay

Doxorubicin is a substrate of P-gp. P-gp-overexpressing CEM/ADR5000 cells were seeded in 12-well plates (10^4^ cells/well). Doxorubicin was obtained from the University Hospital Pharmacy (Mainz, Germany) and used at a concentration of 10 µM in all samples. In parallel, different concentrations of ZINC15675948 (IC_50_, 2 × IC_50_ or 4 × IC_50_) were combined with doxorubicin treatment. Verapamil (20 µM; Sigma-Aldrich) was applied as a positive control for P-gp inhibition. Unstained CEM/ADR5000 cells were used as a negative control. In comparison, non-P-gp-expressing CCRF-CEM cells were used as a positive control for doxorubicin uptake. After 3 h incubation at 37 °C, cells were centrifuged to discard the old medium and resuspended in 1 mL PBS. The doxorubicin fluorescence assay was carried out on a BD Accuri^TM^ C6 Flow Cytometer (Becton-Dickinson) by blue laser at excitation 488 nm and emission 530 nm. In each sample, 3000 cells were counted. Dead cells and debris were removed by gating the cells in forward vs. side scatter, and forward-area vs. forward-height scatter, respectively. All experiments were performed in triplicate. The protocol has been described by us in [74].

MDA-MB-231-pcDNA3 and MDA-MB-BCRP (5000 cells/well) were seeded into 12-well plates. Doxorubicin treatment was combined with ZINC15675948 (IC_50_, 2 × IC_50_ or 4 × IC_50_). Ko143 (200 nM; Sigma-Aldrich) was used as a positive control for BCRP inhibition. The negative control is unstaining cells. Non-BCRP-overexpressing MDA-MB-231-pcDNA cells were applied as a positive control of doxorubicin uptake. After 24-h incubation at 37 °C, the samples were washed and measured as described above.

## 5. Conclusions

In conclusion, ZINC15675948 displayed remarkable cytotoxicity in CCRF-CEM and MDA-MB-231-pcDNA3 cells. ZINC15675948 bound to the c-MYC/MAX interface and inhibited c-MYC activity and expression. The inhibition of c-MYC in MDA-MB-231-pcDNA3 cells involved ubiquitination. By means of microarray-based mRNA expression profiling, we verified that ZINC15675948 induced DNA damage and apoptosis in both cell lines as downstream effects of c-MYC inhibition. ZINC15675948 caused G2/M phase cell cycle arrest in CCRF-CEM cells and S phase arrest in MDA-MB-231-pcDNA3 cells. ZINC15675948 also induced autophagy in CCRF-CEM cells. Furthermore, ZINC15675948 was a substrate of two ABC transporters, P-gp and BCRP. These results illustrate that ZINC15675948 is a promising inhibitor of c-MYC worth further development as a novel anticancer drug.

## Figures and Tables

**Table 1:** Cytotoxicity of ZINC15675948 toward drug-sensitive and -resistant cancer cell lines measured by resazurin reduction assay. CEM/ADR5000 and MDA-MB-BCRP were the two cell lines displaying multidrug-resistant phenotypes by overexpressing P-glycoprotein and BCRP, respectively.

Cell Lines	IC_50_ (µM)	Degree of Resistance
CCRF-CEM	0.008 ± 0.001	8.37
CEM/ADR5000	0.071 ± 0.002	
MDA-MB-231-pcDNA3	0.08 ± 0.004	9
MDA-MB-BCRP	0.72 ± 0.07	

**Table 2:** Molecular docking results of ZINC15675948 and known inhibitors 10058-F4, 10074-A4, and 10075-G5 (positive control) to c-MYC.

Compound	Lowest Binding Energy (kcal/mol)	pKi (µM)	Amino Acids Interactions (Residues in H-Bond Bolded)
ZINC15675948	−9.91	0.055	Arg924, Asp926, **Gln927**, Tyr949, Ile950, Val953
10058-F4	−4.92	247.03 ± 1.5	Arg925, **Gln927**, Pro929, Leu931, Glu932
10074-A4	−6.42 ± 0.01	19.53 ± 0.30	Arg925, **Asp926**, **Gln927**
10074-G5	−6.96 ± 0.01	7.93 ± 0.13	Pro929, **Pro930**, Lys945, Ala948

**Table 3:** In silico molecular docking of ZINC15675948 and control drugs (doxorubicin, verapamil, and Ko143) to P-gp and BCRP.

Protein	Compound	Lowest Binding Energy (kcal/mol)	pKi (µM)	Amino Acids Interactions (Residues in H-Bond Bolded)
P-gp	ZINC15675948	−10.55 ± 0.24	0.07 ± 0.01	Trp232, Phe239, Ala295, Asn296, Ile299, Phe770, **Gln773**, Gly774, Met876, Gln990, Phe994
	Doxorubicin	−6.42 ± 0.05	147.35 ± 72.62	**Ala229**, **Trp232**, **Phe303**, **Ile306**, **Phe343**, **Gln725**, **Ala871**, **Gly872**, **Phe983**, **Met986**, **Ala987**, **Gln990**
	Verapamil	−7.61 ± 0.31	3.0 ± 1.46	Ser228, Ala229, Trp232, Ile306, Phe336, Phe343, Gln725, Phe728, Tyr953, Phe983, Met986, Gln990
BCRP	ZINC15675948	−11.49 ± 0.01	0.007 ± 0.004	A: Leu405, Phe431, Phe432, Thr435, Asn436, Phe439, Thr542, Met549, Leu555 B: Phe432, Thr435, Phe439
	Doxorubicin	−7.00 ± 0.49	122.79 ± 10.96	A: Phe431, Phe432, The435, Asn436, Phe439, Ser440 B: The542, Ile 543, Val546, Met549, Ile555
	Ko143	−10.24 ± 0.19	0.03 ± 0.01	A: Leu405, Phe431, Phe432, Thr435, Asn436, Phe439, Met549, Leu555 B: Phe431, Phe32, **Thr435**, Phe439, Val546, Met549

**Table 4:** The sequence (5′→3′) of qRT-PCR primers.

Gene Symbol	Forward Primer	Reverse Primer
*RAD21*	GAGTCAGCTATGCCTCCACC	TGGAGGTTCTTCTGGGGGAA
*HMGCS1*	CTTTCGTGGCTCACTCCCTT	GTTTCCTCCTTCGGGCACA
*PGK1*	TGTGTGGAATGGTCCTGTGG	TGGCTTTCACCACCTCATCC
*ATP5MF*	CGGACACCAGGACTCCAAAA	GGACTGAAGTCCCGCATCAA
*CITED2*	GGCGAAGCTGGGGAATAACA	AATCAGCCCTCCTCATCCTG
*HSPD1*	GCCGCCCCGCAGAAAT	AAGCCCGAGTGAGATGAGGA
*H4C3*	CAGGGCATTACAAAACCGGC	GTGCTCCGTATAGGTGACGG
*DHFR*	GCCACCGCTCAGGAATGAAT	AGGTTGTGGTCATTCTCTGGAA
*c-MYC*	ACACTAACATCCCACGCTCTG	CTCGCTAAGGCTGGGGAAAG
*GAPDH*	ATGAATGGGCAGCCGTTAGG	AGCATCACCCGGAGGAGAAA

## Data Availability

The authors declare that the data supporting the findings of this study are available within the paper.

## Supplementary Materials

The following supporting information can be downloaded at: https://www.mdpi.com/article/10.3390/molecules28155658/s1, Table S1: Deregulated gene expression upon treatment of CCRF-CEM leukemia cells with the IC_50_ concentration of ZINC15675948 for 24 h; Table S2: Deregulated gene expression upon treatment of MDA-MB-231-pcDNA3 breast cancer cells with the IC_50_ concentration of ZINC15675948 for 24 h.

## Sample Availability

Samples of the compound ZINC15675948 are available upon reasonable request.

## Abbreviations

ABC, ATP-binding cassette transporter; ALL, acute lymphoblastic leukemia; BCRP, breast cancer resistance protein; CDK, cyclin-dependent kinases; DMSO, dimethyl sulfoxide; ELL, eleven-nineteen lysine-rich leukemia gene, elongation factor for RNA polymerase II; FBS, fetal bovine serum; HBSS, Hank’s balanced salt solution; IC_50_, half-maximal inhibitory concentration; IPA, Ingenuity Pathway Analysis; K_d_, dissociation constant; LBE, lowest binding energy; MAX, MYC-associated factor X; MDR, multidrug resistance; MST, microscale thermophoresis; PBS, phosphate buffered saline; P-gp, P-glycoprotein; TNBC, triple-negative breast cancer.

## References

[1] Bishop J.M. (1991). Molecular themes in oncogenesis. Cell.

[2] Dang C.V. (2012). MYC on the path to cancer. Cell.

[3] Lourenco C., Resetca D., Redel C., Lin P., MacDonald A.S., Ciaccio R., Kenney T.M.G., Wei Y., Andrews D.W., Sunnerhagen M. (2021). MYC protein interactors in gene transcription and cancer. Nat. Rev. Cancer.

[4] Albihn A., Johnsen J.I., Henriksson M.A. (2010). MYC in oncogenesis and as a target for cancer therapies. Adv. Cancer Res..

[5] Sammak S., Hamdani N., Gorrec F., Allen M.D., Freund S.M.V., Bycroft M., Zinzalla G. (2019). Crystal structures and nuclear magnetic resonance studies of the apo form of the c-MYC: MAX bHLHZip complex reveal a helical basic region in the absence of DNA. Biochemistry.

[6] Duffy M.J., O’Grady S., Tang M., Crown J. (2021). MYC as a target for cancer treatment. Cancer Treat. Rev..

[7] Gabay M., Li Y., Felsher D.W. (2014). MYC activation is a hallmark of cancer initiation and maintenance. Cold Spring Harb. Perspect. Med..

[8] Llombart V., Mansour M.R. (2022). Therapeutic targeting of “undruggable” MYC. eBioMedicine.

[9] Dhanasekaran R., Deutzmann A., Mahauad-Fernandez W.D., Hansen A.S., Gouw A.M., Felsher D.W. (2022). The MYC oncogene—The grand orchestrator of cancer growth and immune evasion. Nat. Rev. Clin. Oncol..

[10] Sears R.C. (2004). The life cycle of C-myc: From synthesis to degradation. Cell Cycle.

[11] Dingar D., Tu W.B., Resetca D., Lourenco C., Tamachi A., De Melo J., Houlahan K.E., Kalkat M., Chan P.K., Boutros P.C. (2018). MYC dephosphorylation by the PP1/PNUTS phosphatase complex regulates chromatin binding and protein stability. Nat. Commun..

[12] Fallah Y., Brundage J., Allegakoen P., Shajahan-Haq A.N. (2017). MYC-driven pathways in breast cancer subtypes. Biomolecules.

[13] Boxer L.M., Dang C.V. (2001). Translocations involving c-myc and c-myc function. Oncogene.

[14] Felsher D.W., Bishop J.M. (1999). Reversible tumorigenesis by MYC in hematopoietic lineages. Mol. Cell.

[15] Atanasov A.G., Zotchev S.B., Dirsch V.M., Orhan I.E., Banach M., Rollinger J.M., Barreca D., Weckwerth W., Bauer R., Bayer E.A. (2021). Natural products in drug discovery: Advances and opportunities. Nat. Rev. Drug Discov..

[16] Hassan A., Khan A.H., Saleem F., Ahmad H., Khan K.M. (2022). A patent review of pharmaceutical and therapeutic applications of oxadiazole derivatives for the treatment of chronic diseases (2013–2021). Expert Opin. Ther. Pat..

[17] Camci M., Karali N. (2023). Bioisosterism: 1,2,4-oxadiazole rings. ChemMedChem.

[18] Atmaram U.A., Roopan S.M. (2022). Biological activity of oxadiazole and thiadiazole derivatives. Appl. Microbiol. Biotechnol..

[19] Dhameliya T.M., Chudasma S.J., Patel T.M., Dave B.P. (2022). A review on synthetic account of 1,2,4-oxadiazoles as anti-infective agents. Mol. Divers..

[20] Carbone M., Li Y., Irace C., Mollo E., Castelluccio F., Di Pascale A., Cimino G., Santamaria R., Guo Y.-W., Gavagnin M. (2011). Structure and cytotoxicity of phidianidines A and B: First finding of 1,2,4-oxadiazole system in a marine natural product. Org. Lett..

[21] Labriere C., Elumalai V., Staffansson J., Cervin G., Le Norcy T., Denardou H., Réhel K., Moodie L.W.K., Hellio C., Pavia H. (2020). Phidianidine A and synthetic analogues as naturally inspired marine antifoulants. J. Nat. Prod..

[22] Shamsi F., Hasan P., Queen A., Hussain A., Khan P., Zeya B., King H.M., Rana S., Garrison J., Alajmi M.F. (2020). Synthesis and SAR studies of novel 1,2,4-oxadiazole-sulfonamide based compounds as potential anticancer agents for colorectal cancer therapy. Bioorg. Chem..

[23] Caneschi W., Enes K.B., Carvalho de Mendonça C., de Souza Fernandes F., Miguel F.B., da Silva Martins J., Le Hyaric M., Pinho R.R., Duarte L.M., Leal de Oliveira M.A. (2019). Synthesis and anticancer evaluation of new lipophilic 1,2,4 and 1,3,4-oxadiazoles. Eur. J. Med. Chem..

[24] Mohamed M.F.A., Marzouk A.A., Nafady A., El-Gamal D.A., Allam R.M., Abuo-Rahma G.E.-D.A., El Subbagh H.I., Moustafa A.H. (2020). Design, synthesis and molecular modeling of novel aryl carboximidamides and 3-aryl-1,2,4-oxadiazoles derived from indomethacin as potent anti-inflammatory iNOS/PGE2 inhibitors. Bioorg. Chem..

[25] Il’in M.V., Sysoeva A.A., Bolotin D.S., Novikov A.S., Suslonov V.V., Rogacheva E.V., Kraeva L.A., Kukushkin V.Y. (2019). Aminonitrones as highly reactive bifunctional synthons. An expedient one-pot route to 5-amino-1,2,4-triazoles and 5-amino-1,2,4-oxadiazoles—Potential antimicrobials targeting multi-drug resistant bacteria. New J. Chem..

[26] Kim J., Shin J.S., Ahn S., Han S.B., Jung Y.-S. (2018). 3-Aryl-1,2,4-oxadiazole derivatives active against human rhinovirus. ACS Med. Chem. Lett..

[27] Dos Santos Filho J.M., de Queiroz E.S.D.M.A., Macedo T.S., Teixeira H.M.P., Moreira D.R.M., Challal S., Wolfender J.L., Queiroz E.F., Soares M.B.P. (2016). Conjugation of N-acylhydrazone and 1,2,4-oxadiazole leads to the identification of active antimalarial agents. Bioorg. Med. Chem..

[28] Mohammad B.D., Baig M.S., Bhandari N., Siddiqui F.A., Khan S.L., Ahmad Z., Khan F.S., Tagde P., Jeandet P. (2022). Heterocyclic compounds as dipeptidyl peptidase-IV inhibitors with special emphasis on oxadiazoles as potent anti-diabetic agents. Molecules.

[29] Wang M., Liu T., Chen S., Wu M., Han J., Li Z. (2021). Design and synthesis of 3-(4-pyridyl)-5-(4-sulfamido-phenyl)-1,2,4-oxadiazole derivatives as novel GSK-3β inhibitors and evaluation of their potential as multifunctional anti-Alzheimer agents. Eur. J. Med. Chem..

[30] Nelson J.B., Fizazi K., Miller K., Higano C., Moul J.W., Akaza H., Morris T., McIntosh S., Pemberton K., Gleave M. (2012). Phase 3, randomized, placebo-controlled study of zibotentan (ZD4054) in patients with castration-resistant prostate cancer metastatic to bone. Cancer.

[31] Engebraaten O., Vollan H.K.M., Børresen-Dale A.L. (2013). Triple-negative breast cancer and the need for new therapeutic targets. Am. J. Pathol..

[32] Yin L., Duan J.J., Bian X.W., Yu S.C. (2020). Triple-negative breast cancer molecular subtyping and treatment progress. Breast Cancer Res..

[33] Li Q., Pan S., Xie T., Liu H. (2021). MYC in T-cell acute lymphoblastic leukemia: Functional implications and targeted strategies. Blood Sci..

[34] Hegazy M.F., Dawood M., Mahmoud N., Elbadawi M., Sugimoto Y., Klauck S.M., Mohamed N., Efferth T. (2021). 2α-Hydroxyalantolactone from Pulicaria undulata: Activity against multidrug-resistant tumor cells and modes of action. Phytomedicine.

[35] Beaulieu M.E., Soucek L. (2019). Finding MYCure. Mol. Cell. Oncol..

[36] Whitfield J.R., Soucek L. (2021). The long journey to bring a Myc inhibitor to the clinic. J. Cell Biol..

[37] Ghobrial A., Flick N., Daly R., Hoffman M., Milcarek C. (2019). ELL2 influences transcription elongation, splicing, Ig secretion and growth. J. Mucosal Immunol. Res..

[38] Chen Y., Zhou C., Ji W., Mei Z., Hu B., Zhang W., Zhang D., Wang J., Liu X., Ouyang G. (2016). ELL targets c-Myc for proteasomal degradation and suppresses tumour growth. Nat. Commun..

[39] Newman D.J., Cragg G.M. (2020). Natural products as sources of new drugs over the nearly four decades from 01/1981 to 09/2019. J. Nat. Prod..

[40] Yap J.L., Wang H., Hu A., Chauhan J., Jung K.Y., Gharavi R.B., Prochownik E.V., Fletcher S. (2013). Pharmacophore identification of c-Myc inhibitor 10074-G5. Bioorg. Med. Chem. Lett..

[41] Hammoudeh D.I., Follis A.V., Prochownik E.V., Metallo S.J. (2009). Multiple independent binding sites for small-molecule inhibitors on the oncoprotein c-Myc. J. Am. Chem. Soc..

[42] Massó-Vallés D., Soucek L. (2020). Blocking Myc to treat cancer: Reflecting on two decades of omomyc. Cells.

[43] Han H., Jain A.D., Truica M.I., Izquierdo-Ferrer J., Anker J.F., Lysy B., Sagar V., Luan Y., Chalmers Z.R., Unno K. (2019). Small-molecule MYC inhibitors suppress tumor growth and enhance immunotherapy. Cancer Cell.

[44] Boike L., Cioffi A.G., Majewski F.C., Co J., Henning N.J., Jones M.D., Liu G., McKenna J.M., Tallarico J.A., Schirle M. (2021). Discovery of a functional covalent ligand targeting an intrinsically disordered cysteine within MYC. Cell Chem. Biol..

[45] Panda D., Saha P., Das T., Dash J. (2017). Target guided synthesis using DNA nano-templates for selectively assembling a G-quadruplex binding c-MYC inhibitor. Nat. Commun..

[46] Michel J., Cuchillo R. (2012). The impact of small molecule binding on the energy landscape of the intrinsically disordered protein C-myc. PLoS ONE.

[47] Follis A.V., Hammoudeh D.I., Wang H., Prochownik E.V., Metallo S.J. (2008). Structural rationale for the coupled binding and unfolding of the c-Myc oncoprotein by small molecules. Chem. Biol..

[48] Farrell A.S., Sears R.C. (2014). MYC degradation. Cold Spring Harb. Perspect. Med..

[49] Chen Y., Sun X.-X., Sears R.C., Dai M.-S. (2019). Writing and erasing MYC ubiquitination and SUMOylation. Genes Dis..

[50] Lu Y., Liu Y., Yang C. (2017). Evaluating In Vitro DNA Damage Using Comet Assay. J. Vis. Exp..

[51] Goga A., Yang D., Tward A.D., Morgan D.O., Bishop J.M. (2007). Inhibition of CDK1 as a potential therapy for tumors over-expressing MYC. Nat. Med..

[52] Gu Y., Rosenblatt J., Morgan D.O. (1992). Cell cycle regulation of CDK2 activity by phosphorylation of Thr160 and Tyr15. EMBO J..

[53] Kurbegovic A., Trudel M. (2020). The master regulators Myc and p53 cellular signaling and functions in polycystic kidney disease. Cell. Signal..

[54] Kelly G.L., Grabow S., Glaser S.P., Fitzsimmons L., Aubrey B.J., Okamoto T., Valente L.J., Robati M., Tai L., Fairlie W.D. (2014). Targeting of MCL-1 kills MYC-driven mouse and human lymphomas even when they bear mutations in p53. Genes Dev..

[55] Li X., He S., Ma B. (2020). Autophagy and autophagy-related proteins in cancer. Mol. Cancer.

[56] Kang R., Zeh H.J., Lotze M.T., Tang D. (2011). The Beclin 1 network regulates autophagy and apoptosis. Cell Death Differ..

[57] Liu W.J., Ye L., Huang W.F., Guo L.J., Xu Z.G., Wu H.L., Yang C., Liu H.F. (2016). p62 links the autophagy pathway and the ubiqutin–proteasome system upon ubiquitinated protein degradation. Cell Mol. Biol. Lett..

[58] Elbadawi M., Boulos J.C., Dawood M., Zhou M., Gul W., ElSohly M.A., Klauck S.M., Efferth T. (2023). The novel artemisinin dimer isoniazide ELI-XXIII-98-2 induces c-MYC inhibition, DNA damage, and autophagy in leukemia cells. Pharmaceutics.

[59] Li W., Zhang H., Assaraf Y.G., Zhao K., Xu X., Xie J., Yang D.H., Chen Z.S. (2016). Overcoming ABC transporter-mediated multidrug resistance: Molecular mechanisms and novel therapeutic drug strategies. Drug Resist. Updates.

[60] Robey R.W., Pluchino K.M., Hall M.D., Fojo A.T., Bates S.E., Gottesman M.M. (2018). Revisiting the role of ABC transporters in multidrug-resistant cancer. Nat. Rev. Cancer.

[61] Tiwari A.K., Sodani K., Dai C.L., Ashby C.R., Chen Z.S. (2011). Revisiting the ABCs of multidrug resistance in cancer chemotherapy. Curr. Pharm. Biotechnol..

[62] Doyle L., Ross D.D. (2003). Multidrug resistance mediated by the breast cancer resistance protein BCRP (ABCG2). Oncogene.

[63] Silva R., Vilas-Boas V., Carmo H., Dinis-Oliveira R.J., Carvalho F., de Lourdes Bastos M., Remião F. (2015). Modulation of P-glycoprotein efflux pump: Induction and activation as a therapeutic strategy. Pharmacol. Ther..

[64] Szakács G., Paterson J.K., Ludwig J.A., Booth-Genthe C., Gottesman M.M. (2006). Targeting multidrug resistance in cancer. Nat. Rev. Drug Discov..

[65] Modi A., Roy D., Sharma S., Vishnoi J.R., Pareek P., Elhence P., Sharma P., Purohit P. (2022). ABC transporters in breast cancer: Their roles in multidrug resistance and beyond. J. Drug Target..

[66] Hall M.D., Handley M.D., Gottesman M.M. (2009). Is resistance useless? Multidrug resistance and collateral sensitivity. Trends Pharmacol. Sci..

[67] Efferth T., Konkimalla V.B., Wang Y.F., Sauerbrey A., Meinhardt S., Zintl F., Mattern J., Volm M. (2008). Prediction of broad spectrum resistance of tumors towards anticancer drugs. Clin. Cancer Res..

[68] Saeed M.E.M., Boulos J.C., Elhaboub G., Rigano D., Saab A., Loizzo M.R., Hassan L.E.A., Sugimoto Y., Piacente S., Tundis R. (2019). Cytotoxicity of cucurbitacin E from Citrullus colocynthis against multidrug-resistant cancer cells. Phytomedicine.

[69] Saeed M.E.M., Mahmoud N., Sugimoto Y., Efferth T., Abdel-Aziz H. (2018). Molecular determinants of sensitivity or resistance of cancer cells toward sanguinarine. Front. Pharmacol..

[70] Doyle L.A., Yang W., Abruzzo L.V., Krogmann T., Gao Y., Rishi A.K., Ross D.D. (1998). A multidrug resistance transporter from human MCF-7 breast cancer cells. Proc. Natl. Acad. Sci. USA.

[71] Kadioglu O., Cao J., Kosyakova N., Mrasek K., Liehr T., Efferth T. (2016). Genomic and transcriptomic profiling of resistant CEM/ADR-5000 and sensitive CCRF-CEM leukaemia cells for unravelling the full complexity of multi-factorial multidrug resistance. Sci. Rep..

[72] Efferth T., Sauerbrey A., Olbrich A., Gebhart E., Rauch P., Weber H.O., Hengstler J.G., Halatsch M.E., Volm M., Tew K.D. (2003). Molecular modes of action of artesunate in tumor cell lines. Mol. Pharmacol..

[73] Kimmig A., Gekeler V., Neumann M., Frese G., Handgretinger R., Kardos G., Diddens H., Niethammer D. (1990). Susceptibility of multidrug-resistant human leukemia cell lines to human interleukin 2-activated killer cells. Cancer Res..

[74] Abdelfatah S., Böckers M., Asensio M., Kadioglu O., Klinger A., Fleischer E., Efferth T. (2021). Isopetasin and S-isopetasin as novel P-glycoprotein inhibitors against multidrug-resistant cancer cells. Phytomedicine.

[75] O’Brien J., Wilson I., Orton T., Pognan F. (2000). Investigation of the Alamar Blue (resazurin) fluorescent dye for the assessment of mammalian cell cytotoxicity. Eur. J. Biochem..

[76] Yin X., Giap C., Lazo J.S., Prochownik E.V. (2003). Low molecular weight inhibitors of Myc-Max interaction and function. Oncogene.

[77] Kallio M.A., Tuimala J.T., Hupponen T., Klemelä P., Gentile M., Scheinin I., Koski M., Käki J., Korpelainen E.I. (2011). Chipster: User-friendly analysis software for microarray and other high-throughput data. BMC Genom..

[78] Zhou M., Boulos J.C., Klauck S.M., Efferth T. (2023). The cardiac glycoside ZINC253504760 induces parthanatos-type cell death and G_2_/M arrest via downregulation of MEK1/2 phosphorylation in leukemia cells. Cell Biol. Toxicol..

[79] Livak K.J., Schmittgen T.D. (2001). Analysis of relative gene expression data using real-time quantitative PCR and the 2^−ΔΔCT^ method. Methods.

[80] Benhusein G.M., Mutch E., Aburawi S., Williams F.M. (2010). Genotoxic effect induced by hydrogen peroxide in human hepatoma cells using comet assay. Libyan J. Med..

[81] Gyori B.M., Venkatachalam G., Thiagarajan P.S., Hsu D., Clement M.V. (2014). OpenComet: An automated tool for comet assay image analysis. Redox Biol..

[82] Collins A.R. (2004). The comet assay for DNA damage and repair. Mol. Biotechnol..

[83] Crowley L.C., Marfell B.J., Scott A.P., Waterhouse N.J. (2016). Quantitation of apoptosis and necrosis by annexin V binding, propidium iodide uptake, and flow cytometry. Cold Spring Harb. Protoc..

[84] Shynkar V.V., Klymchenko A.S., Kunzelmann C., Duportail G., Muller C.D., Demchenko A.P., Freyssinet J.-M., Mely Y. (2007). Fluorescent biomembrane probe for ratiometric detection of apoptosis. J. Am. Chem. Soc..

