# Supplementary material for: Modes of Action of a Novel c-MYC Inhibiting 1,2,4-Oxadiazole Derivative in Leukemia and Breast Cancer Cells
Source: Molecules. 2023 Jul 26;28(15):5658. doi: 10.3390/molecules28155658 (PMC10419799; doi:10.3390/molecules28155658)
Supplement: Supplementary file 1 [file molecules-28-05658-s001.zip › molecules-2482907-supplementary.pdf]

Supplementary Tables

Modes of action of a novel c-MYC inhibiting 1,2,4-oxadiazole derivative in leukemia and breast cancer cells

Min Zhou[1], Joelle C. Boulos[1], Ejlal A. Omer[1], Sabine M. Klauck[2], Thomas Efferth[1]

[1] Department of Pharmaceutical Biology, Institute of Pharmaceutical and Biomedical Sciences, Johannes Gutenberg University-Mainz, Staudinger Weg 5, 55128 Mainz, Germany

[2] Division of Cancer Genome Research, German Cancer Research Center (DKFZ), German Cancer Consortium (DKTK), National Center for Tumor Disease (NCT), Im Neuenheimer Feld 460, 69120 Heidelberg, Germany

*** Correspondence to:** Prof. Dr. Thomas Efferth, E-mail: efferth@uni-mainz.de

Supplementary Table S1: Deregulated gene expression upon treatment of CCRF-CEM leukemia cells with the IC_{50} concentration of ZINC15675948 for 24 h.

Symbol	Gene name	Fold change of expression
PGK1	Phosphoglycerate kinase 1	-894.824
H2BC15	H2B-clustered histone 15	-874.55
NREP	Neuronal regeneration-related protein	-782.619
ATP5MF	ATP synthase membrane subunit f	-774.632
ATP5MC1	ATP synthase membrane subunit c locus 1	-623.959
ANXA2	Annexin A2	-609.842
GPI	Glucose-6-phosphate isomerase	-585.909
RPL36A	Ribosomal protein L36a	-564.794
H2AC14	H2A-clustered histone 14	-558.444
STARD7	StAR-related lipid transfer domain containing 7	-554.385
MBTPS1	Membrane-bound transcription factor peptidase, site 1	-466.755
MYC	MYC proto-oncogene, bHLH transcription factor	-436.409
AASDHPPT	Aminoadipate-semialdehyde dehydrogenase-phosphopantetheinyl transferase	-423.601
SNU13	Small nuclear ribonucleoprotein 13	-401.352
DNAJC7	DnaJ heat shock protein family (Hsp40) member C7	-391.026
TIMM13	Translocase of inner mitochondrial membrane 13	-390.194
PSMD6	Proteasome 26S subunit, non-ATPase 6	-380.012
POLR3H	RNA polymerase III subunit H	-378.485
SKP2	S-phase kinase-associated protein 2	-376.452
ATP5ME	ATP synthase membrane subunit e	-371.18
PSMC4	Proteasome 26S subunit, ATPase 4	-345.009
UQCRH	Ubiquinol-cytochrome c reductase hinge protein	-330.192
SLIRP	SRA stem loop-interacting RNA-binding protein	-330.171
POLR2L	RNA polymerase II, I and III subunit L	-315.839
FH	Fumarate hydratase	-315.692
POLR2J	RNA polymerase II subunit J	-312.908
H3C1	H3-clustered histone 1	-289.204
SGTA	Small glutamine rich tetratricopeptide repeat co-chaperone α	-287.344
GGCT	γ-Glutamylcyclotransferase	-277.642
MED21	Mediator complex subunit 21	-276.1
TOMM5	Translocase of outer mitochondrial membrane 5	-264.689
RBM4	RNA binding motif protein 4	-260.937
ARPC4	Actin-related protein 2/3 complex subunit 4	-256.843
PRMT5	Protein arginine methyltransferase 5	-255.082
NOP10	NOP10 ribonucleoprotein	-242.713
IMP3	IMP U3 small nucleolar ribonucleoprotein 3	-241.662
HIGD1A	HIG1 hypoxia-inducible domain family member 1A	-238.727
MBD2	Methyl-CpG binding domain protein 2	-236.407
GLO1	Glyoxalase I	-234.15
PRPF3	Pre-mRNA processing factor 3	-232.382
MCRS1	Microspherule protein 1	-228.699
GIMAP6	GTPase, IMAP family member 6	-227.072
BCL7B	BAF chromatin remodeling complex subunit BCL7B	-222.003
TIAL1	TIA1 cytotoxic granule-associated RNA binding protein-like 1	-216.678
POLR2B	RNA polymerase II subunit B	-212.393

AKT2	AKT serine/threonine kinase 2	-205.971
SLC39A14	Solute carrier family 39 member 14	-204.958
TSR2	TSR2 ribosome maturation factor	-204.401
VPS26C	VPS26 endosomal protein sorting factor C	-201.543
SLC38A5	Solute carrier family 38 member 5	-199.146
CDC23	Cell division cycle 23	-196.17
SNAP29	Synaptosome-associated protein 29	-194.53
PRCC	Proline-rich mitotic checkpoint control factor	-194.1
TXNDC17	Thioredoxin domain-containing 17	-193.404
TFB2M	Transcription factor B2, mitochondrial	-192.639
ECD	Ecdysoneless cell cycle regulator	-191.991
ESS2	Ess-2 splicing factor homolog	-190.523
NOP14	NOP14 nucleolar protein	-189.579
FBL	Fibrillarin	-185.399
PRKCSH	Protein kinase C substrate 80K-H	-178.751
TIMM22	Translocase of inner mitochondrial membrane 22	-174.109
CLEC11A	C-type lectin domain-containing 11A	-171.233
BOP1	BOP1 ribosomal biogenesis factor	-170.653
SENP3	SUMO specific peptidase 3	-162.169
HMGN3	High mobility group nucleosomal binding domain 3	-158.5
TARS1	Threonyl-tRNA synthetase 1	-150.394
PLOD1	Procollagen-lysine,2-oxoglutarate 5-dioxygenase 1	-150.187
PRX	Periaxin	-146.378
RIPK1	Receptor-interacting serine/threonine kinase 1	-145.21
MIS12	MIS12 kinetochore complex component	-144.341
NAPA	NSF attachment protein alpha	-140.725
CCT6A	Chaperonin containing TCP1 subunit 6A	-140.677
SYPL1	Synaptophysin-like 1	-140.605
ABRAXAS2	Abraxas 2, BRISC complex subunit	-140.3
IKZF5	IKAROS family zinc finger 5	-139.584
PPP1R10	Protein phosphatase 1 regulatory subunit 10	-137.914
HSP90AB1	Heat shock protein 90 alpha family class B member 1	-136.445
CHKA	Choline kinase α	-135.811
CHRAC1	Chromatin accessibility complex subunit 1	-132.085
NCBP1	Nuclear cap binding protein subunit 1	-131.961
IFT22	Intraflagellar transport 22	-131.708
USP5	Ubiquitin-specific peptidase 5	-130.705
ZNF672	Zinc finger protein 672	-129.878
BCL11A	BAF chromatin remodeling complex subunit BCL11A	-127.513
UBR7	Ubiquitin protein ligase E3 component n-recognin 7	-125.692
SPRING1	SREBF pathway regulator in Golgi 1	-125.591
ATG16L1	Autophagy-related 16-like 1	-125.555
MAPKAPK2	MAPK-activated protein kinase 2	-124.144
ST7	Suppression of tumorigenicity 7	-121.841
GPN2	GPN-loop GTPase 2	-121.82
TOE1	Target of EGR1, exonuclease	-120.285
BCL9	BCL9 transcription coactivator	-119.233
TSPAN7	Tetraspanin 7	-116.64
SLX1A-SULT1A3	SLX1A-SULT1A3 readthrough (NMD candidate)	-115.898
CHID1	Chitinase domain-containing 1	-112.854

ANKS6	Ankyrin repeat and sterile α motif domain-containing 6	-112.21
FOXO3	Forkhead box O3	-111.784
ELP6	Elongator acetyltransferase complex subunit 6	-110.671
DNM2	Dynamin 2	-110.671
CD151	CD151 molecule (Raph blood group)	-110.061
PMM2	Phosphomannomutase 2	-108.248
LOC107987373	39S ribosomal protein L23, mitochondrial	-108.088
TOR1AIP2	Torsin 1A-interacting protein 2	-107.437
SFXN5	Sideroflexin 5	-106.865
SLC35B4	Solute carrier family 35 member B4	-106.326
TMEM104	Transmembrane protein 104	-106.045
DHX15	DEAH-box helicase 15	-101.834
RNF185	Ring finger protein 185	-101.731
ZDHHC7	Zinc finger DHHC-type palmitoyltransferase 7	-99.753
AP3B1	Adaptor-related protein complex 3 subunit β 1	-99.31
PUS1	Pseudouridine synthase 1	-99.052
BRMS1	BRMS1 transcriptional repressor and anoikis regulator	-97.909
FBLN2	Fibulin 2	-97.847
ZNF317	Zinc finger protein 317	-97.834
TMEM185A	Transmembrane protein 185A	-96.499
POLR3K	RNA polymerase III subunit K	-95.872
HYOU1	Hypoxia-upregulated 1	-95.303
AFMID	Arylformamidase	-95.271
ABCF2	ATP-binding cassette subfamily F member 2	-94.311
RAI1	Retinoic acid-induced 1	-93.834
TNFRSF14-AS1	TNFRSF14 antisense RNA 1	-93.019
STK32C	Serine/threonine kinase 32C	-91.713
HS6ST1	Heparan sulfate 6-O-sulfotransferase 1	-90.21
AHDC1	AT-hook DNA binding motif-containing 1	-89.51
KIF3B	Kinesin family member 3B	-87.485
CSRNP2	Cysteine- and serine-rich nuclear protein 2	-86.996
ASF1B	Anti-silencing function 1B histone chaperone	-85.769
PRR3	Proline-rich 3	-85.707
SF3B3	Splicing factor 3b subunit 3	-85.667
ANAPC2	Anaphase-promoting complex subunit 2	-85.008
AMH	Anti-Mullerian hormone	-84.671
ARHGAP27	Rho GTPase-activating protein 27	-84.02
B4GALNT1	β-1,4-N-acetyl-galactosaminyltransferase 1	-83.727
ZFYVE27	Zinc finger FYVE-type-containing 27	-83.455
FBXO9	F-box protein 9	-83.212
ADCY3	Adenylate cyclase 3	-82.795
PTGDR2	Prostaglandin D2 receptor 2	-81.399
TXNL4A	Thioredoxin like 4A	-81.187
ELOF1	Elongation factor 1 homolog	-79.966
EXOSC10	Exosome component 10	-76.678
L3MBTL2	L3MBTL histone methyl-lysine binding protein 2	-74.95
SLC25A39	Solute carrier family 25 member 39	-74.914
IQSEC1	IQ motif and Sec7 domain ArfGEF 1	-74.844
CDKN2B	Cyclin-dependent kinase inhibitor 2B	-72.444
WRN	WRN RecQ-like helicase	71.17

NAPB	NSF attachment protein β	71.336
FAM8A1	Family with sequence similarity 8 member A1	72.726
LINC00869	Long intergenic non-protein coding RNA 869	73.366
EFCAB2	EF-hand calcium-binding domain 2	73.697
U2AF1L4	U2 small nuclear RNA auxiliary factor 1-like 4	74.23
CSGALNACT2	Chondroitin sulfate N-acetylgalactosaminyltransferase 2	74.263
MAN1A1	Mannosidase α class 1A member 1	74.305
LCN1	Lipocalin 1	75.07
CACFD1	Calcium channel flower domain-containing 1	76.61
CD38	CD38 molecule	76.717
ENGASE	Endo-β-N-acetylglucosaminidase	78.287
TMEM168	Transmembrane protein 168	78.56
LCMT1	Leucine carboxyl methyltransferase 1	78.723
NR6A1	Nuclear receptor subfamily 6 group A member 1	79.988
STN1	STN1 subunit of CST complex	81.089
ANKRD20A4P	Ankyrin repeat domain 20 family member A3, pseudogene	81.324
HEATR6	HEAT repeat-containing 6	81.344
TMEM30A	Transmembrane protein 30A	81.702
BORA	BORA aurora kinase A activator	83.845
SH3GLB1	SH3 domain-containing GRB2-like, endophilin B1	84.86
REEP5	Receptor accessory protein 5	85.025
HMGCL	3-Hydroxy-3-methylglutaryl-CoA lyase	85.573
TNFAIP8	TNFα-induced protein 8	85.886
SLC14A1	Solute carrier family 14 member 1 (Kidd blood group)	85.971
METTL22	Methyltransferase like 22	85.982
MARCKS	Myristoylated alanine-rich protein kinase C substrate	86.112
MACIR	Macrophage immunometabolism regulator	86.192
LRRC20	Leucine-rich repeat-containing 20	87.422
RAB44	RAB44, member RAS oncogene family	89.843
FANCF	FA complementation group F	90.573
SUPT20HL2	SUPT20H-like 2	91.542
PPP2R5A	Protein phosphatase 2 regulatory subunit B'α	91.69
DENND3	DENN domain-containing 3	91.813
DDX60	DExD/H-box helicase 60	93.808
STX16-NPEPL1	STX16-NPEPL1 readthrough (NMD candidate)	93.87
CXorf65	Chromosome X open reading frame 65	94.625
WDR27	WD repeat domain 27	95.287
MCUR1	Mitochondrial calcium uniporter regulator 1	95.382
IL10RB	Interleukin 10 receptor subunit β	97.649
HMG20A	High mobility group 20A	97.722
ABHD10	Abhydrolase domain-containing 10, depalmitoylase	97.887
CPSF2	Cleavage- and polyadenylation-specific factor 2	98.271
SUGP2	SURP and G-patch domain-containing 2	98.564
GCLM	Glutamate-cysteine ligase modifier subunit	98.944
BAZ1A	Bromodomain adjacent to zinc finger domain 1A	99.427
STIL	STIL centriolar assembly protein	100.531
PTP4A3	Protein tyrosine phosphatase 4A3	101.141
C16orf87	Chromosome 16 open reading frame 87	101.263
ABHD13	Abhydrolase domain-containing 13	101.298
NACA	Nascent polypeptide-associated complex subunit α	101.743

TENT2	Terminal nucleotidyltransferase 2	103.956
TMBIM4	Transmembrane BAX inhibitor motif-containing 4	103.958
TMEM87B	Transmembrane protein 87B	104.158
MED14	Mediator complex subunit 14	105.199
MLH1	mutL homolog 1	105.427
HCFC2	Host cell factor C2	106.566
RIMKLB	Ribosomal modification protein rimK-like family member B	106.743
SNX16	Sorting nexin 16	106.933
DPP8	Dipeptidyl peptidase 8	107.017
CYSLTR1	Cysteinyl leukotriene receptor 1	107.203
FOXN2	Forkhead box N2	107.26
RASA1	RAS p21 protein activator 1	108.176
DOCK11	Dedicator of cytokinesis 11	109.51
FAM193A	Family with sequence similarity 193 member A	111.941
EXPH5	Exophilin 5	112.988
HLCS	Holocarboxylase synthetase	114.492
SEC24A	SEC24 homolog A, COPII coat complex component	114.939
USP9X	Ubiquitin specific peptidase 9 X-linked	115.068
PRKCI	Protein kinase C ι	117.58
INTS12	Integrator complex subunit 12	117.858
KANK3	KN motif and ankyrin repeat domains 3	118.255
SPG11	SPG11 vesicle trafficking-associated, spatacsin	118.942
FAHD2A	Fumarylacetoacetate hydrolase domain-containing 2A	119.981
ABHD18	Abhydrolase domain-containing 18	120.788
LYRM7	LYR motif-containing 7	122.308
CCNT1	Cyclin T1	124.051
SUMO2	Small ubiquitin-like modifier 2	124.082
CENPC	Centromere protein C	124.282
TATDN3	TatD DNase domain-containing 3	124.482
AP1S2	Adaptor-elated protein complex 1 subunit σ 2	124.79
GIT2	GIT ArfGAP 2	125.219
TPD52	Tumor protein D52	125.326
PPIE	Peptidylprolyl isomerase E	127.464
NLN	Neurolysin	129.273
ZNF816-ZNF321P	ZNF816-ZNF321P readthrough	129.648
EDEM3	ER degradation enhancing α-mannosidase-like protein 3	130.569
EIF5A2	Eukaryotic translation initiation factor 5A2	131.707
MSH5	mutS homolog 5	132.54
PDCD4	Programmed cell death 4	133.13
TRIM59	Tripartite motif-containing 59	134.562
UCHL3	Ubiquitin C-terminal hydrolase L3	135.375
SPINT2	Serine peptidase inhibitor, Kunitz-type 2	138.421
MIS18A	MIS18 kinetochore protein A	138.69
NCK1	NCK adaptor protein 1	138.925
TMEM106B	Transmembrane protein 106B	139.132
SDHAF4	Succinate dehydrogenase complex assembly factor 4	140.279
CAST	Calpastatin	141.739
NFATC2	Nuclear factor of activated T cells 2	145.285
RAB8B	RAB8B, member RAS oncogene family	146.196

ARHGAP5	Rho GTPase activating protein 5	147.266
LRRC8D	Leucine-rich repeat-containing 8 VRAC subunit D	147.869
MARCHF7	Membrane associated ring CH-type finger 7	148.89
MNAT1	MNAT1 component of CDK activating kinase	149.97
SAMD9	Sterile α motif domain-containing 9	151.835
EXOSC8	Exosome component 8	152.27
EIF3A	Eukaryotic translation initiation factor 3 subunit A	153.724
SPIN1	Spindlin 1	153.82
FHIP2A	FHF complex subunit HOOK-interacting protein 2A	154.286
NEK2	NIMA-related kinase 2	157.207
AIDA	Axin interactor, dorsalization-associated	157.22
MSGN1	Mesogenin 1	159.898
ZNF280D	Zinc finger protein 280D	161.461
MTFMT	Mitochondrial methionyl-tRNA formyltransferase	162.111
ARPC3	Actin-related protein 2/3 complex subunit 3	163.55
DDX50	DExD-box helicase 50	165.167
NPEPL1	Aminopeptidase-like 1	165.386
PICALM	Phosphatidylinositol-binding clathrin assembly protein	165.577
CD53	CD53 molecule	168.629
GINM1	Glycoprotein integral membrane 1	169.79
EPRS1	Glutamyl-prolyl-tRNA synthetase 1	170.902
ELMO2	Engulfment and cell motility 2	171.041
MOCS2	Molybdenum cofactor synthesis 2	172.192
LNPEP	Leucyl and cystinyl aminopeptidase	174.1
C5orf22	Chromosome 5 open reading frame 22	174.924
CLPX	Caseinolytic mitochondrial matrix peptidase chaperone subunit X	176.265
ZRANB2	Zinc finger RANBP2-type-containing 2	177.924
COG6	Component of oligomeric Golgi complex 6	178.308
RTN4	Reticulon 4	185.407
CNIH1	Cornichon family AMPA receptor auxiliary protein 1	185.821
COPB1	COPI coat complex subunit β 1	191.712
TARBP1	TAR (HIV-1) RNA-binding protein 1	194.064
LCE1C	Late cornified envelope 1C	204.749
TUBG2	Tubulin γ 2	205.397
TCAIM	T cell activation inhibitor, mitochondrial	211.119
DFFA	DNA fragmentation factor subunit α	211.338
SEL1L3	SEL1L family member 3	213.03
PHF20	PHD finger protein 20	213.052
OSGIN2	Oxidative stress-induced growth inhibitor family member 2	213.386
GOLGA8T	Golgin A8 family member T	216.317
FAM86B1	Family with sequence similarity 86 member B1	219.993
HCLS1	Hematopoietic cell-specific Lyn substrate 1	220.254
MDM4	MDM4 regulator of p53	220.422
IFRD1	Interferon-related developmental regulator 1	224.495
SH2D1A	SH2 domain-containing 1A	225.236
AIMP1	Aminoacyl tRNA synthetase complex-interacting multifunctional protein 1	226.88
CCNB1IP1	Cyclin B1-interacting protein 1	235.862
PIAS1	Protein inhibitor of activated STAT 1	236.804

CDKN3	Cyclin-dependent kinase inhibitor 3	239.766
ARRDC3	Arrestin domain-containing 3	243.376
BLZF1	Basic leucine zipper nuclear factor 1	248.504
ARFGAP3	ADP ribosylation factor GTPase-activating protein 3	250.63
HDGF	Heparin-binding growth factor	262.287
IBTK	Inhibitor of Bruton tyrosine kinase	262.403
RNPEP	Arginyl aminopeptidase	264.07
CHST11	Carbohydrate sulfotransferase 11	266.767
PGRMC2	Progesterone receptor membrane component 2	268.332
PRKRA	Protein activator of interferon-induced protein kinase EIF2AK2	277.993
TUT7	Terminal uridylyl transferase 7	283.322
TSG101	Tumor susceptibility 101	285.937
VIM	Vimentin	290.791
CCDC88A	Coiled-coil domain-containing 88A	302.785
IMPA1	Inositol monophosphatase 1	309.746
H2AZ2	H2A.Z variant histone 2	315.471
TMEM179B	Transmembrane protein 179B	315.863
IFI27L1	Interferon α inducible protein 27-like 1	327.252
ICA1	Islet cell autoantigen 1	338.459
TUBE1	Tubulin ε 1	339.814
CLDND1	Claudin domain-containing 1	344.32
UBA3	Ubiquitin-like modifier-activating enzyme 3	361.52
SNRPA1	Small nuclear ribonucleoprotein polypeptide A'	401.878
CD3D	CD3d molecule	405.906
THAP9-AS1	THAP9 antisense RNA 1	414.691
PTBP3	Polypyrimidine tract binding protein 3	423.094
USP11	Ubiquitin-specific peptidase 11	445.03
ZEB1	Zinc finger E-box-binding homeobox 1	475.977
TPT1	Tumor protein, translationally-controlled 1	529.088
SNX5	Sorting nexin 5	558.026
HSPA9	Heat shock protein family A (Hsp70) member 9	561.504
IDI1	Isopentenyl-diphosphate delta isomerase 1	624.931
SNHG32	Small nucleolar RNA host gene 32	728.082
ASNS	Asparagine synthetase (glutamine-hydrolyzing)	864.481
RAD21	RAD21 cohesin complex component	978.419
TMBIM6	Transmembrane BAX inhibitor motif-containing 6	983.752
HMGCS1	3-hydroxy-3-methylglutaryl-CoA synthase 1	1187.922

Supplementary Table S2: Deregulated gene expression upon treatment of MDA-MB-231-pcDNA3 breast cancer cells with the IC_{50} concentration of ZINC15675948 for 24 h.

Symbol	Gene name	Fold change of expression
H4C3	H4-clustered histone 3	-1097.46
CALR	Calreticulin	-786.152
TOP2A	DNA topoisomerase II α	-747.107
H2AC13	H2A-clustered histone 13	-707.585
ARL6IP1	ADP ribosylation factor-like GTPase 6-interacting protein 1	-648.657
DHFR	Dihydrofolate reductase	-545.609
H2BC18	H2B-clustered histone 18	-506.868
UBC	Ubiquitin C	-503.295
SCD	Stearoyl-CoA desaturase	-496.162
MYH9	Myosin heavy chain 9	-495.637
LAMB3	Laminin subunit β 3	-489.462
H4C11	H4-clustered histone 11	-468.663
PLAU	Plasminogen activator, urokinase	-434.661
CSE1L	Chromosome segregation 1-like	-425.176
CBX5	Chromobox 5	-420.682
FAM83D	Family with sequence similarity 83 member D	-410.028
CTSB	Cathepsin B	-398.833
MCL1	MCL1 apoptosis regulator, BCL2 family member	-388.41
MORF4L1	Mortality factor 4-like 1	-361.289
DPY19L1	Dpy-19-like C-mannosyltransferase 1	-359.667
RAB31	RAB31, member RAS oncogene family	-349.893
ADGRF5	Adhesion G protein-coupled receptor F5	-346.867
TUBB4A	Tubulin β 4A class IVa	-343.719
SLC30A9	Solute carrier family 30 member 9	-328.453
DUT	Deoxyuridine triphosphatase	-327.3
FBL	Fibrillarin	-325.331
NUCKS1	Nuclear casein kinase and cyclin-dependent kinase substrate 1	-324.544
SAMHD1	SAM and HD domain-containing deoxynucleoside triphosphate triphosphohydrolase 1	-310.46
CPNE8	Copine 8	-300.725
LTA4H	Leukotriene A4 hydrolase	-288.132
HYOU1	Hypoxia-upregulated 1	-264.062
SPRY1	Sprouty RTK signaling antagonist 1	-258.877
DNMBP	Dynamin-binding protein	-252.625
PDIA6	Protein disulfide isomerase family A member 6	-251.66
SURF4	Surfeit 4	-247.503
MOB1A	MOB kinase activator 1A	-246.226
GALNT10	Polypeptide N-acetylgalactosaminyltransferase 10	-234.075
ABCD3	ATP-binding cassette subfamily D member 3	-233.045
PTBP1	Polypyrimidine tract binding protein 1	-228.707
SLCO4C1	Solute carrier organic anion transporter family member 4C1	-224.322
ARHGAP29	Rho GTPase-activating protein 29	-220.073
UACA	Uveal autoantigen with coiled-coil domains and ankyrin repeats	-217.66

YWHAB	Tyrosine 3-monooxygenase/tryptophan 5-monooxygenase activation protein β	-217.607
STK17A	Serine/threonine kinase 17a	-215.483
PRPF8	Pre-mRNA-processing factor 8	-210.087
STAU2	Staufen double-stranded RNA binding protein 2	-206.656
WEE1	WEE1 G2 checkpoint kinase	-199.398
KNL1	Kinetochore scaffold 1	-198.563
CRIPT	CXXC repeat containing interactor of PDZ3 domain	-197.78
ATP5F1C	ATP synthase F1 subunit γ	-188.806
SRSF6	Serine- and arginine-rich splicing factor 6	-184.144
SORT1	Sortilin 1	-182.36
POGLUT3	Protein O-glucosyltransferase 3	-180.049
TMTC3	Transmembrane O-mannosyltransferase targeting cadherins 3	-178.812
CENPI	Centromere protein I	-175.693
TMEM156	Transmembrane protein 156	-169.921
FDPS	Farnesyl diphosphate synthase	-168.581
AKT2	AKT serine/threonine kinase 2	-164.96
GOLIM4	Golgi integral membrane protein 4	-161.132
DIAPH3	Diaphanous-related formin 3	-158.056
RFC3	Replication factor C subunit 3	-156.621
EDEM3	ER degradation enhancing α-mannosidase-like protein 3	-156.105
CHRAC1	Chromatin accessibility complex subunit 1	-155.619
MAGI1	Membrane-associated guanylate kinase, WW and PDZ domain-containing 1	-154.971
POLR2K	RNA polymerase II, I and III subunit K	-154.954
AMIGO2	Adhesion molecule with Ig like domain 2	-149.367
TGOLN2	Trans-Golgi network protein 2	-143.607
LNPEP	Leucyl and cystinyl aminopeptidase	-142.927
PLOD2	Procollagen-lysine,2-oxoglutarate 5-dioxygenase 2	-141.146
PYGB	Glycogen phosphorylase B	-138.181
CD151	CD151 molecule (Raph blood group)	-136.351
MED31	Mediator complex subunit 31	-135.623
RAB10	RAB10, member RAS oncogene family	-128.945
ASPM	Assembly factor for spindle microtubules	-128.634
H2BC15	H2B-clustered histone 15	-128.102
TRIM25	Tripartite motif-containing 25	-127.719
ANKRD36C	Ankyrin repeat domain 36C	-126.06
GFPT2	Glutamine-fructose-6-phosphate transaminase 2	-125.366
SLC37A4	Solute carrier family 37 member 4	-120.177
DSG2	Desmoglein 2	-118.543
ABR	ABR activator of RhoGEF and GTPase	-110.982
LIN7C	Lin-7 homolog C, crumbs cell polarity complex component	-108.677
PARP14	Poly(ADP-ribose) polymerase family member 14	-107.404
FZD6	Frizzled class receptor 6	-106.219
PCGF3	Polycomb group ring finger 3	-106.034
HAP1	Huntingtin-associated protein 1	-104.383
CHRNA5	Cholinergic receptor nicotinic α 5 subunit	-102.973
SNX14	Sorting nexin 14	-100.057
GPD2	Glycerol-3-phosphate dehydrogenase 2	-96.261
PIK3R2	Phosphoinositide-3-kinase regulatory subunit 2	-96.17

MND1	Meiotic nuclear divisions 1	-96.055
MED16	Mediator complex subunit 16	-95.558
CASD1	CAS1 domain-containing 1	-87.454
RASSF7	Ras association domain family member 7	-86.107
DCAF7	DDB1- and CUL4-associated factor 7	-85.957
ENY2	ENY2 transcription and export complex 2 subunit	-85.938
SMC1A	Structural maintenance of chromosomes 1A	-79.591
CPNE7	Copine 7	-78.336
DNAJC4	DnaJ heat shock protein family (Hsp40) member C4	-77.829
TMEM185B	Transmembrane protein 185B	-77.068
EPHB2	EPH receptor B2	-76.939
FBXL17	F-box and leucine-rich repeat protein 17	-76.847
SPC25	SPC25 component of NDC80 kinetochore complex	-76.43
KAT2B	Lysine acetyltransferase 2B	-76.299
DDX58	DExD/H-box helicase 58	-75.809
MICU2	Mitochondrial calcium uptake 2	-75.698
DAP	Death-associated protein	-74.386
ASF1B	Anti-silencing function 1B histone chaperone	-73.008
HGSNAT	Heparan-α-glucosaminide N-acetyltransferase	-71.105
ZNF780B	Zinc finger protein 780B	-70.864
EXT2	Exostosin glycosyltransferase 2	-70.778
ZNF91	Zinc finger protein 91	-70.133
USP48	Ubiquitin-specific peptidase 48	-69.95
FKBP9	FKBP prolyl isomerase 9	-69.34
KLF2	Kruppel-like factor 2	-68.74
DDX17	DEAD-box helicase 17	-68.192
LY6E	Lymphocyte antigen 6 family member E	-67.501
SIPA1L1	Signal-induced proliferation-associated 1-like 1	-67.432
SLC25A1	Solute carrier family 25 member 1	-67.186
DHRS11	Dehydrogenase/reductase 11	-67.075
BAD	BCL2-associated agonist of cell death	-63.458
CCNF	Cyclin F	-61.653
CYP2R1	Cytochrome P450 family 2 subfamily R member 1	-61.464
PCNX3	Pecanex 3	-60.807
KIF15	Kinesin family member 15	-60.387
H3C3	H3-clustered histone 3	-60.076
SENP2	SUMO-specific peptidase 2	-59.953
BMPR1A	Bone morphogenetic protein receptor type 1A	-59.627
DSN1	DSN1 component of MIS12 kinetochore complex	-59.349
TST	Thiosulfate sulfurtransferase	-59.232
SURF1	SURF1 cytochrome c oxidase assembly factor	-58.863
MTA3	Metastasis-associated 1 family member 3	-58.763
APMAP	Adipocyte plasma membrane-associated protein	-58.116
B3GALT6	β-1,3-galactosyltransferase 6	-55.707
EIF5A2	Eukaryotic translation initiation factor 5A2	55.297
ELOF1	Elongation factor 1 homolog	55.803
FDX2	Ferredoxin 2	55.901
H2BC4	H2B-clustered histone 4	57.311
ZNF35	Zinc finger protein 35	58.327
TPM2	Tropomyosin 2	58.432

DDB2	Damage-specific DNA binding protein 2	58.899
TMEM39A	Transmembrane protein 39A	59.07
FNIP2	Folliculin-interacting protein 2	59.609
TRIP12	Thyroid hormone receptor interactor 12	60.762
LGR6	Leucine-rich repeat containing G protein-coupled receptor 6	61.377
TMEM170A	Transmembrane protein 170A	61.808
CDT1	Chromatin licensing and DNA replication factor 1	62.022
INPP5D	Inositol polyphosphate-5-phosphatase D	62.074
GRAMD1B	GRAM domain-containing 1B	62.997
SUPT6H	SPT6 homolog, histone chaperone and transcription elongation factor	63.263
EPS8L2	EPS8-like 2	63.862
NKIRAS1	NFκB inhibitor-interacting Ras-like 1	64.995
NEK3	NIMA-related kinase 3	65.678
TSPAN12	Tetraspanin 12	65.83
RMND1	Required for meiotic nuclear division 1 homolog	65.836
TENT4B	Terminal nucleotidyltransferase 4B	66.663
ZKSCAN8	Zinc finger with KRAB and SCAN domains 8	67.119
NABP1	Nucleic acid-binding protein 1	67.159
C9orf78	Chromosome 9 open reading frame 78	67.299
ZNF224	Zinc finger protein 224	67.643
TRMU	tRNA mitochondrial 2-thiouridylase	67.75
MFSD14A	Major facilitator superfamily domain-containing 14A	68.092
ELAPOR2	Endosome-lysosome-associated apoptosis and autophagy regulator family member 2	68.205
ZNF830	Zinc finger protein 830	69.262
ERN1	Endoplasmic reticulum to nucleus signaling 1	69.698
PRRG1	Proline-rich and Gla domain 1	69.737
MAGEA3/MAGEA6	MAGE family member A6	70.045
POLR1E	RNA polymerase I subunit E	70.147
THUMPD3	THUMP domain-containing 3	70.912
METTL26	Methyltransferase-like 26	71.309
RNF138	Ring finger protein 138	71.744
ATG14	Autophagy-related 14	74.14
FTSJ1	FtsJ RNA 2'-O-methyltransferase 1	75.094
CBFA2T2	CBFA2/RUNX1 partner transcriptional co-repressor 2	75.199
VCPKMT	Valosin-containing protein lysine methyltransferase	76.015
WDR70	WD repeat domain 70	76.199
RPIA	Ribose 5-phosphate isomerase A	77.126
GAL	Galanin and GMAP prepropeptide	77.201
AADAT	Aminoadipate aminotransferase	78.975
NR6A1	Nuclear receptor subfamily 6 group A member 1	80.068
CLK4	CDC-like kinase 4	80.353
CLNS1A	Chloride nucleotide-sensitive channel 1A	80.517
HSBP1L1	Heat shock factor binding protein 1-like 1	81.27
POLR1H	RNA polymerase I subunit H	81.528
ATXN7	Ataxin 7	82.02
POLR1D	RNA polymerase I and III subunit D	84.109
FEM1C	Fem-1 homolog C	85.033

GSTM1	Glutathione S-transferase μ 1	85.645
XPNPEP3	X-Prolyl aminopeptidase 3	86.14
ZBTB43	Zinc finger and BTB domain-containing 43	86.175
RPL26L1	Ribosomal protein L26-like 1	86.38
SREK1IP1	SREK1-interacting protein 1	86.863
RBPMS	RNA-binding protein, mRNA-processing factor	86.998
CCT6A	Chaperonin-containing TCP1 subunit 6A	87.047
TM4SF19	Transmembrane 4 L six family member 19	87.855
COIL	Coilin	89.488
NR3C1	Nuclear receptor subfamily 3 group C member 1	89.823
RRN3	RRN3 homolog, RNA polymerase I transcription factor	89.962
MDK	Midkine	91.067
POU2F1	POU class 2 homeobox 1	92.917
ZKSCAN1	Zinc finger with KRAB and SCAN domains 1	93.135
SHOC2	SHOC2 leucine-rich repeat scaffold protein	93.549
YIPF5	Yip1 domain family member 5	93.577
STX3	Syntaxin 3	94.238
SLU7	SLU7 homolog, splicing factor	94.321
MBNL2	Muscleblind-like splicing regulator 2	94.64
DSP	Desmoplakin	94.944
RNF8	Ring finger protein 8	95.095
CEP95	Centrosomal protein 95	95.906
UGCG	UDP-glucose ceramide glucosyltransferase	96.844
VAMP1	Vesicle-associated membrane protein 1	97.016
RNF7	Ring finger protein 7	98.758
INSYN2B	Inhibitory synaptic factor family member 2B	98.804
KIAA1191	KIAA1191	99.37
THG1L	tRNA-histidine guanylyltransferase 1-like	99.45
KRTAP4-8	Keratin-associated protein 4-8	100.498
RBBP6	RB-binding protein 6, ubiquitin ligase	102.24
ZKSCAN5	Zinc finger with KRAB and SCAN domains 5	102.778
ING1	Inhibitor of growth family member 1	105.506
AOX1	Aldehyde oxidase 1	105.814
IFIT3	Interferon-induced protein with tetratricopeptide repeats 3	106.53
NBPF10	NBPF member 19	108.136
MAP3K20	Mitogen-activated protein kinase kinase kinase 20	108.245
PAXX	PAXX non-homologous end joining factor	108.657
BRAP	BRCA1-associated protein	109.161
HTATIP2	HIV-1 Tat interactive protein 2	109.472
ETV3	ETS variant transcription factor 3	109.553
LIMCH1	LIM and calponin homology domains 1	110.228
SFSWAP	Splicing factor SWAP	110.251
FHL2	Four-and-a-half LIM domains 2	111.968
CCDC80	Coiled-coil domain-containing 80	112.731
F3	Coagulation factor III, tissue factor	114.255
BLZF1	Basic leucine zipper nuclear factor 1	114.674
MTHFD1L	Methylenetetrahydrofolate dehydrogenase (NADP+ dependent) 1-like	114.975
IGFBP3	Insulin like growth factor binding protein 3	116.779
RIT1	Ras-like without CAAX 1	116.814

MED13L	Mediator complex subunit 13L	117.062
ASS1	Argininosuccinate synthase 1	119.328
HEXIM1	HEXIM P-TEFb complex subunit 1	122.322
CCBE1	Collagen- and calcium-binding EGF domains 1	122.652
S100A3	S100 calcium-binding protein A3	125.082
MMP1	Matrix metallopeptidase 1	127.808
EIF1B	Eukaryotic translation initiation factor 1B	128.681
PYCR2	Pyrroline-5-carboxylate reductase 2	129.312
STAG3L2/STAG3L3	Stromal antigen 3-like 3 (pseudogene)	129.46
PCBD2	Pterin-4 α-carbinolamine dehydratase 2	129.723
RGS10	Regulator of G protein signaling 10	130.062
ZNF143	Zinc finger protein 143	130.254
ARFGEF2	ADP ribosylation factor guanine nucleotide exchange factor 2	132.626
ABL2	ABL proto-oncogene 2, non-receptor tyrosine kinase	134.526
RBM18	RNA binding motif protein 18	136.216
SDC4	Syndecan 4	140.876
ALYREF	Aly/REF export factor	140.923
ACTN1	Actinin α 1	142.456
ENOPH1	Enolase-phosphatase 1	143.155
SLC35F2	Solute carrier family 35 member F2	144.051
DCUN1D5	Defective in cullin neddylation 1 domain-containing 5	144.226
PAQR3	Progestin and adipoQ receptor family member 3	146.382
UCK2	Uridine cytidine kinase 2	148.743
ASNS	Asparagine synthetase (glutamine-hydrolyzing)	148.99
TIMM13	Translocase of inner mitochondrial membrane 13	152.287
RNF6	Ring finger protein 6	152.845
LCORL	Ligand dependent nuclear receptor corepressor-like	154.168
VAT1	Vesicle amine transport 1	154.932
NPIPB15	Nuclear pore complex-interacting protein family member B8	155.586
S100A4	S100 calcium binding protein A4	158.592
SMNDC1	Survival motor neuron domain-containing 1	160.593
KLHL15	Kelch-like family member 15	160.805
ARID3A	AT-rich interaction domain 3A	162.426
WSB1	WD repeat and SOCS box-containing 1	163.953
GPBP1L1	GC-rich promoter binding protein 1-like 1	165.591
CPEB4	Cytoplasmic polyadenylation element binding protein 4	168.095
SRSF9	Serine- and arginine-rich splicing factor 9	168.402
CASP4	Caspase 4	168.811
UFC1	Ubiquitin-fold modifier-conjugating enzyme 1	172.373
PPP4R3B	Protein phosphatase 4 regulatory subunit 3B	173.181
ELL2	Elongation factor for RNA polymerase II 2	173.38
GATA6	GATA-binding protein 6	175.096
SYT1	Synaptotagmin 1	176.42
EIF3G	Eukaryotic translation initiation factor 3 subunit G	176.556
APOLD1	Apolipoprotein L domain-containing 1	176.767
KIDINS220	Kinase D-interacting substrate 220	179.522
GDF15	Growth differentiation factor 15	181.344
ARF1	ADP ribosylation factor 1	184.859
ZFAND3	Zinc finger AN1-type-containing 3	188.983
EXOC2	Exocyst complex component 2	192.435

SARS1	Seryl-tRNA synthetase 1	194.348
LYAR	Ly1 antibody reactive	195.187
PRMT1	Protein arginine methyltransferase 1	202.335
RBM14-RBM4	RBM14-RBM4 readthrough	212.244
YBX1	Y-box binding protein 1	212.354
H3-3A/H3-3B	H3.3 histone A	217.032
SRGN	Serglycin	222.049
GLRX5	Glutaredoxin 5	222.452
SNHG32	Small nucleolar RNA host gene 32	225.827
TGIF2-RAB5IF	TGIF2-RAB5IF readthrough	227.207
NKTR	Natural killer cell-triggering receptor	243.194
SNRPA1	Small nuclear ribonucleoprotein polypeptide A'	253.328
SLIRP	SRA stem loop-interacting RNA-binding protein	259.866
EIF3D	Eukaryotic translation initiation factor 3 subunit D	278.56
KCTD5	Potassium channel tetramerization domain-containing 5	280.39
GARS1	Glycyl-tRNA synthetase 1	281.754
MDH2	Malate dehydrogenase 2	287.648
NIFK	Nucleolar protein interacting with the FHA domain of MKI67	296.404
AURKA	Aurora kinase A	300.635
YBX3	Y-box binding protein 3	318.446
EIF3H	Eukaryotic translation initiation factor 3 subunit H	350.246
SNAPC1	Small nuclear RNA-activating complex polypeptide 1	356.776
RPL18	Ribosomal protein L18	402.44
UBR4	Ubiquitin protein ligase E3 component n-recognin 4	407.523
SQSTM1	Sequestosome 1	422.613
NIP7	Nucleolar pre-rRNA-processing protein NIP7	474.22
CITED2	Cbp/p300 interacting transactivator with Glu/Asp-rich carboxy-terminal domain 2	566.464
HSPD1	Heat shock protein family D (Hsp60) member 1	600.082
